# Mechanical‐medical convergence in heart failure: Artificial intelligence, finite‐element modeling, and 3D printing for diagnosis and prognosis

**DOI:** 10.1002/btm2.70156

**Published:** 2026-06-18

**Authors:** Quazi Noor E Sabrina, Quazi Md Zobaer Shah, Quazi Noor E Sohela, Md Mahabub Hasan Mousum, Md. Moyeen Uddin Chisty, Quazi Md. Akbar Shah

**Affiliations:** ^1^ Department of Virology Dhaka Medical College and Hospital Dhaka Bangladesh; ^2^ Nuclear Safety Division Bangladesh Atomic Energy Regulatory Authority Dhaka Bangladesh; ^3^ Institute of Statistical Research and Training University of Dhaka Dhaka Bangladesh; ^4^ Department of Mechanical & Aerospace Engineering Old Dominion University Norfolk Virginia USA; ^5^ Emergency & Casualty Department Government Employees Hospital Dhaka Bangladesh; ^6^ Small Arms Factory Bangladesh Ordnance Factories Gazipur Bangladesh

**Keywords:** 3D printing/additive manufacturing, artificial intelligence (AI)/machine learning, digital twin, finite‐element modeling, heart failure (HF), precision cardiology, ventricular remodeling

## Abstract

Heart failure remains a leading cause of global morbidity and mortality, yet routine clinical indices often miss the regional biomechanical disturbances that drive progression and shape treatment response. This State‐of‐the‐Art review examines how finite‐element (FE) modeling, additive manufacturing, and artificial intelligence (AI) are converging to improve the diagnosis, phenotyping, procedural planning, and prognostic assessment of heart failure (HF). Although these technologies have matured in structural heart disease and transcatheter intervention research, their greatest translational potential may lie in HF, where patient‐specific ventricular remodeling, myocardial stress–strain heterogeneity, valve‐ventricular coupling, and device‐tissue interaction are incompletely captured by conventional clinical indices. We synthesize translational and clinical literature published from 2015 to 2026 on imaging‐derived FE models, multimaterial 3D‐printed cardiovascular phantoms, and AI‐based analytic pipelines relevant to HF populations or HF‐related decision pathways. FE approaches provide mechanistic estimates of regional myocardial deformation, wall stress, and remodeling trajectories; 3D‐printed phantoms enable bench‐top validation, device rehearsal, and hemodynamic replication; and AI supports segmentation, phenotyping, multimodal integration, and risk prediction. Across all three domains, however, the evidence base remains dominated by retrospective studies, bench validation, and small translational cohorts, with limited prospective outcome validation and inconsistent reporting of reproducibility. We therefore propose an evidence‐aware, HF‐centered framework for integrating computational mechanics, physical phantoms, and data‐driven models into digital‐physical twin workflows. From a clinical perspective, the near‐term opportunity is not autonomous decision‐making, but better mechanistic phenotyping, more transparent procedural planning, and more rigorous testing of patient‐specific hypotheses before intervention. Standardized reporting, external validation, careful materials characterization, and multicenter endpoint‐linked studies will be essential for clinical translation.

## Graphical abstract

As summarized in the Graphical Abstract, we propose a digital‐physical twin framework tailored to heart failure, in which patient‐specific clinical and imaging data inform finite‐element‐based computational models of cardiac mechanics. These digital twins are complemented by multimaterial 3D‐printed physical phantoms that allow experimental validation and procedural rehearsal. Artificial intelligence facilitates scalable implementation through automated segmentation, feature extraction, and surrogate modeling. Critically, model outputs are iteratively calibrated against benchtop measurements and clinical heart failure endpoints, enabling mechanophenotype‐based risk stratification and therapy planning beyond conventional metrics such as ejection fraction alone. Central Illustration. Mechanical‐Medical Convergence for Precision Heart Failure Care. Patient‐specific heart failure data, including multimodality imaging, hemodynamics, electrical information, and clinical markers, are integrated to construct a computational (digital) twin using finite‐element cardiac mechanics. These models quantify regional stress, strain, wall tension, and remodeling risk beyond conventional metrics. A complementary physical twin, generated through multimaterial 3D printing, enables benchtop testing, device rehearsal, and experimental validation. Artificial intelligence supports automated image segmentation, feature extraction, and surrogate modeling to accelerate analysis. A dedicated validation and calibration loop links computational predictions with bench testing and clinical outcomes, including heart failure hospitalization, mortality, and therapy response. This integrated framework supports mechanophenotype‐driven diagnosis, prognosis, and individualized therapy planning in heart failure.

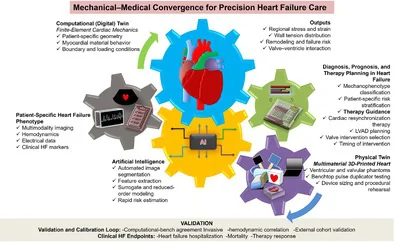

**Translational Impact Statement**

Integrating imaging‐derived finite‐element cardiac mechanics, multimaterial 3D‐printed patient phantoms, and data‐driven models enables a translational digital–physical twin approach that links mechanistic insight with experimental validation and predictive analytics; this framework can improve patient‐specific diagnosis, procedural planning, and risk stratification, streamline device testing, and accelerate clinical translation of precision heart‐failure interventions.

## 1 INTRODUCTION

Heart failure (HF) remains a major global health challenge, affecting more than 64 million individuals worldwide and contributing to substantial mortality, frequent hospitalizations, and escalating socioeconomic burden.^1^, ^2^ Despite advances in pharmacological therapy, device‐based interventions, and surgery, clinical outcomes in HF remain highly variable. This variability reflects the complex, patient‐specific interactions among myocardial remodeling, valvular dysfunction, and ventricular–vascular coupling that govern disease progression and response to therapy.^3^, ^4^, ^5^ Conventional diagnostic approaches, which rely largely on global indices such as left ventricular ejection fraction, chamber dimensions, and surrogate pressure‐volume metrics, often fail to capture the underlying biomechanical abnormalities that precede irreversible dysfunction or determine therapeutic efficacy.^6^

At its core, HF is a disease of disturbed cardiac mechanics. Alterations in myocardial material properties, wall stress distribution, fiber architecture, and ventricular geometry drive maladaptive remodeling and progressive functional decline.^7^ Valvular heart disease (VHD) frequently coexists with HF, either as a primary driver through chronic pressure or volume overload or as a secondary consequence of ventricular dilation and annular deformation.^8^ Although anatomical imaging and Doppler‐based hemodynamic assessment remain central to clinical practice, these methods provide limited insight into regional stress–strain behavior, energetic inefficiency, and early biomechanical derangements that may precede overt functional deterioration.^9^

Finite‐element (FE) modeling has emerged as a powerful framework for quantifying cardiac mechanics by integrating imaging‐derived geometry, constitutive myocardial behavior, and physiologically relevant boundary conditions.^10^ Early studies demonstrated the ability of FE models to estimate regional myocardial stress and predict remodeling under pressure‐ and volume‐overload states, establishing a mechanistic basis for understanding HF progression.^6^ Advances in cardiac magnetic resonance (CMR) and three‐dimensional (3D) echocardiography have further enabled increasingly patient‐specific FE models, narrowing the translational gap between computational biomechanics and clinical cardiology.^6^

In parallel, 3D printing has gained prominence in cardiovascular medicine, initially for anatomical visualization and procedural planning.^11^ In VHD, patient‐specific printed phantoms have been widely used to simulate transcatheter valve deployment, assess paravalvular leakage, and support training in anatomically complex scenarios.^12^ More recently, the integration of FE‐derived stress and deformation fields with 3D printing has enabled the fabrication of functional cardiac phantoms that reproduce both anatomy and mechanical behavior, allowing experimental validation of computational predictions through in vitro testing.^13^

Recent cardiovascular artificial intelligence (AI) literature highlights the value of integrating data‐driven learning with physics‐based digital twins to improve interpretability and scalability, while also emphasizing that most current approaches remain retrospectively validated and insufficiently mature for unsupervised clinical decision support.^14^, ^15^, ^16^, ^17^, ^18^ However, most AI‐driven HF applications remain largely phenomenological and insufficiently linked to underlying myocardial and valvular mechanics, raising concerns regarding interpretability, generalizability, and clinical trust.^19^ Recent conceptual advances therefore emphasize the convergence of FE modeling, 3D printing, and AI into integrated “digital‐physical twin” paradigms, in which mechanistic modeling, experimental validation, and data‐driven prediction inform patient‐specific care.^10^

Although promising, most studies to date are retrospective, include modest sample sizes, and rely on surrogate endpoints, underscoring the need for outcome‐linked validation. Despite their promise, adoption of these technologies in routine HF management is constrained by nonstandardized workflows, limited validation against longitudinal clinical outcomes, a relative scarcity of HF‐focused studies compared with valve‐centric applications, and unresolved regulatory and ethical challenges related to AI‐enabled decision support and advanced manufacturing.^14^, ^19^, ^20^ A comparison of recent review articles highlights this gap and underscores the distinct HF‐centric focus of the present work (Table 1).

**TABLE 1:** Comparison of recent review articles on finite‐element modeling, artificial intelligence, and three‐dimensional printing in heart valve and heart failure research.

Review (Year)	Context/focus	Methods/tech	Highlights/notes
Wang et al. (2021)^12^	Structural heart interventions (e.g., TAVR/TMVR)	3D Printing, Computational Modeling (FE), AI	Overview of 3D‐printed models and simulations for transcatheter valve procedures; links imaging to simulation and training. Focus on procedural planning and physician training, not HF‐centric.
Ma et al. (2021)^21^	Cardiac 3D printing applications (CHD, valve disease)	3D Printing	Systematic review of 3D printing in various heart diseases (CHD, TAVR, hypertrophic CM); illustrates clinical cases (e.g., TAVR training models). Does not cover AI or FEM.
Haddad et al. (2025)^20^	Valvular replacement (bioprinted valves)	3D Bioprinting, AI	Survey of 3D bioprinted, patient‐specific valve prostheses and AI‐driven design; emphasizes tissue‐engineering solutions for VHD. Novel focus on bioprinting plus AI for anticoagulant‐free valves.
Bamford et al. (2024)^22^	Valvular heart disease (diagnosis & therapy)	AI	State‐of‐the‐art review of AI for VHD: auscultation, ECG/Echo interpretation, and intervention planning. Highlights AI in imaging and clinical decision‐making; no 3D printing or FEM.
Park MH et al. (2021)^23^	Heart valve biomechanics (repair & mechanics)	In silico (FE), Ex vivo	Reviews computational simulations and ex vivo heart simulators for valve mechanics. Emphasizes biomechanical insights (annulus motion, strain) and experimental flow loops. Focus on biomechanics, not AI or printing.
Agarwal A et al. (2025)^24^	Structural heart simulation (TAVR, TMVR, LAAO)	Computational Simulation (FEA, CFD, FSI)	Covers patient‐specific finite‐element and fluid dynamics models for multiple interventions. Discusses simulation platforms and suggests integrating machine learning; does not address 3D printing explicitly.
Present review	HF‐centered translational synthesis	FE, 3D printing, AI	Focuses on HF pathophysiology, evidence maturity, and digital‐physical twin integration.

This review was conducted as a narrative, targeted literature synthesis rather than a formal systematic review. We searched PubMed and Scopus for peer‐reviewed publications from January 2015 through February 2026 and supplemented database searching with backward citation tracking of key original articles and relevant review papers. Search terms combined controlled vocabulary and free‐text keywords related to HF and the enabling technologies discussed in this review, including “HF,” “cardiac remodeling,” “FE,” “biomechanics,” “3D printing,” “additive manufacturing,” “AI,” “machine learning (ML),” “digital twin,” “ventricular assist device (VAD),” “transcatheter,” and “patient‐specific model.” Titles and abstracts were screened for relevance to HF pathophysiology, HF‐related procedural planning, patient‐specific cardiovascular modeling, and translational validation. We prioritized studies that directly addressed HF populations, informed HF‐related decision pathways, or provided enabling methods later applied to HF‐relevant structural heart applications. We excluded noncardiovascular applications, purely technical engineering studies without cardiovascular interpretive relevance, duplicate records, and superseded reports. Additional seminal and widely cited studies were included where necessary to provide historical context and conceptual completeness.

## 2 CARDIAC BIOMECHANICS AND FE MODELING

In HF, FE modeling is particularly relevant for quantifying regional stress patterns that drive ventricular remodeling and functional decline. While many technical advances in FE and structural heart modeling originate in valve and device research, this review focuses on how these computational and physical modeling techniques inform HF care. Mechanically driven processes—including regional wall stress, adverse remodeling, valve–ventricular coupling, and device–tissue interaction—are central to HF progression and therapeutic response. Accordingly, the following sections interpret each methodology in terms of its relevance to HF phenotyping, clinical decision‐making, and level of outcome validation. Within this HF‐centered framework, bioprosthetic valve mechanics, and structural valve degeneration are considered primarily in terms of their impact on ventricular loading conditions, remodeling trajectories, and overall ventricular–vascular coupling.

### 2.1 Core HF pathophysiology as the translational anchor

HF is fundamentally driven by multiscale alterations spanning myocardial structure, mechanics, and systemic hemodynamics. Central to this process is ventricular remodeling, characterized by changes in chamber geometry, wall stress distribution, and contractile efficiency, often accompanied by myocardial fibrosis that alters tissue stiffness and electromechanical coupling. In parallel, electrical and mechanical dyssynchrony further impairs coordinated contraction, reducing pump efficiency and promoting adverse remodeling. Distinct HF phenotypes, particularly heart failure with preserved ejection fraction (HFpEF), are increasingly recognized as disorders of diastolic dysfunction, microvascular impairment, and ventricular–vascular uncoupling rather than isolated systolic failure. Importantly, ventricular–vascular coupling governs the interaction between cardiac contractility and arterial load, serving as a critical determinant of energetic efficiency and clinical progression. These interconnected mechanisms provide the physiological foundation upon which computational modeling, data‐driven approaches, and experimental platforms must be interpreted to ensure translational relevance. Conditions including obesity, diabetes mellitus, metabolic syndrome, and hypertension contribute to a chronic systemic inflammatory state marked by increased circulating cytokine levels, dysregulated adipokine profiles, and endothelial activation. At the cellular level, dysregulation of calcium handling contributes to divergent phenotypes of heart failure with reduced ejection fraction (HFrEF) and HFpEF, influencing both systolic contractility and diastolic relaxation dynamics. A most recent review, as shown in Figure 1 further highlights those alterations in calcium‐regulating proteins—including SERCA2a, RyR2, and the Na^+^/Ca^2+^ exchanger—lead to impaired calcium cycling, thereby linking molecular dysfunction to macroscopic features such as ventricular remodeling, stiffness, and impaired relaxation. These findings reinforce the multiscale nature of HF pathophysiology and support the integration of cellular mechanisms into computational and translational modeling frameworks.

**FIGURE 1:**
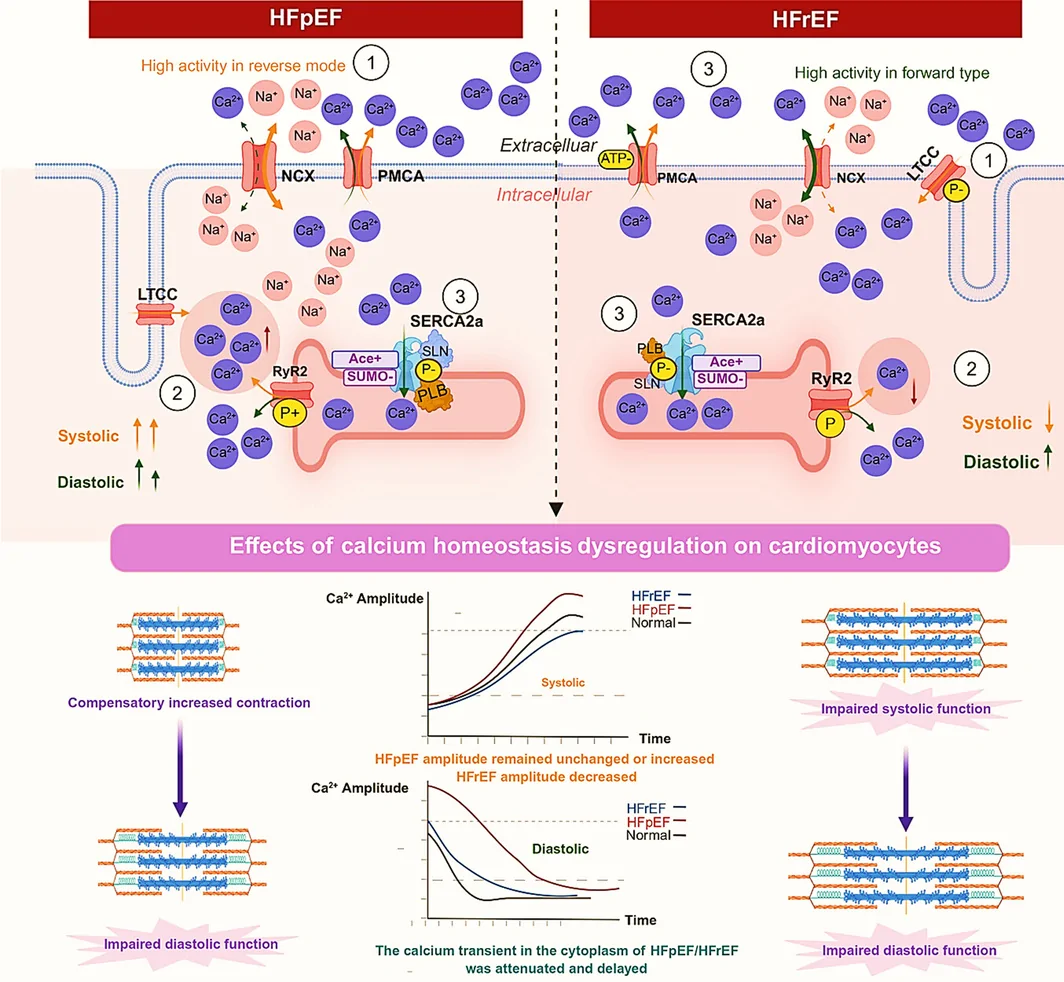
Pathological differences in calcium handling between heart failure with reduced ejection fraction (HFrEF) and heart failure with preserved ejection fraction (HFpEF). At the molecular level, key calcium‐regulating proteins—including SERCA2a, RyR2, Na^+^/Ca^2+^ exchanger (NCX), plasma membrane Ca^2+^ ATPase (PMCA), and L‐type Ca^2+^ channels (LTCC)—exhibit altered expression and function, leading to impaired calcium cycling. At the functional level, these changes affect calcium transients and sarcomeric dynamics during systole and diastole, contributing to impaired contractility in HFrEF and diastolic dysfunction in HFpEF. Reproduced from 25 under the terms of the Creative Commons Attribution 4.0 International License (CC BY 4.0).

Because this review is centered on HF, the valve‐focused examples below are included only where they alter ventricular loading conditions, contribute to HF‐relevant structural deterioration, or provide transferable computational and experimental methods for mechanistic phenotyping, intervention planning, and device–tissue interaction.

### 2.2 FE modeling of valve mechanics and HF–relevant biomechanics

Bioprosthetic heart valves (BHVs) remain widely used because they provide favorable hemodynamics, no audible mechanical noise, and less need for lifelong anticoagulation than mechanical valves.^26^, ^27^ Their major limitation is reduced durability, as structural valve deterioration arises from both biologically mediated tissue degeneration and calcification and mechanically driven fatigue under repetitive cyclic loading.^28^, ^29^, ^30^, ^31^, ^32^ These processes often interact, making valve longevity and failure patterns difficult to predict.^26^, ^27^, ^33^, ^34^

Under cyclic loading, BHV leaflets undergo progressive geometric and microstructural remodeling. After approximately 50 million cycles, corresponding to roughly 2 years of in vivo function, leaflets show permanent deformation, especially in the central belly region, as illustrated in Figure 2 (left).^26^ This remodeling is accompanied by collagen redistribution rather than overt fiber rupture, and collagen orientation metrics remain relatively stable despite substantial macroscopic deformation.^35^, ^36^ Accordingly, BHV fatigue is often viewed as a staged process in which early geometric remodeling precedes stress redistribution, microstructural injury, and eventual leaflet failure.^37^

**FIGURE 2:**
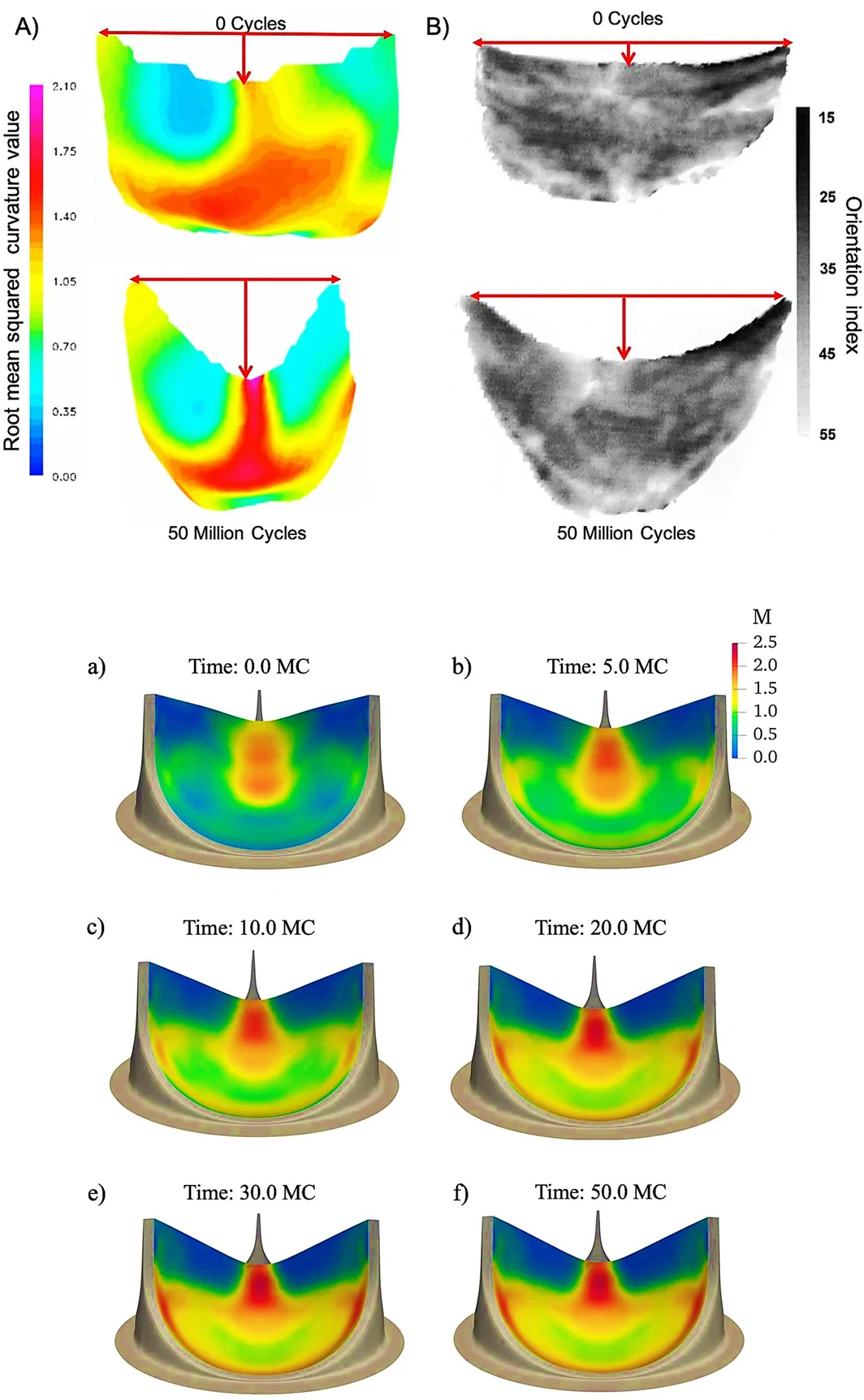
Fatigue‐induced geometric remodeling (left) and deformation evolution in bioprosthetic heart valve (BHV) leaflets (right). Representative deformation patterns of BHV leaflets following prolonged cyclic loading demonstrate permanent geometric remodeling localized primarily within the leaflet belly region. Concurrently, fatigue‐associated plastic deformation leads to progressive alterations in leaflet curvature and deformation metrics over repeated loading cycles. Together, these observations illustrate the spatial and temporal evolution of leaflet mechanics during fatigue progression. Adapted from 26 (with permission, License Number 6238580974023).

Time‐dependent FE frameworks have therefore been developed to model this long‐term biomechanical evolution.^26^, ^37^ These approaches use constitutive laws for exogenously crosslinked soft tissues to simulate changing leaflet geometry, material properties, and collagen architecture under prolonged cyclic loading. A typical workflow includes quasi‐static simulation of the initial valve, updating of material parameters to account for plastic deformation, and iterative geometric remeshing across loading cycles. Compared with uncycled valves, valves subjected to 50 million cycles show reduced circumferential and radial stretch, reflecting progressive stiffening and more homogeneous deformation patterns.^26^ These changes, illustrated in Figure 2 (right), demonstrate the spatial and temporal evolution of stretch, shear, and curvature during fatigue.

Collagen fiber architecture plays a central role in governing the mechanical response and fatigue behavior of BHVs. Comparative FE simulations incorporating isotropic fiber distributions, bovine pericardial fiber architectures, and native porcine aortic valve fiber architectures demonstrate pronounced differences in strain heterogeneity and deformation patterns.^26^, ^38^

As shown in Figure 3, valves with isotropic or exogenously crosslinked pericardial fiber architectures exhibit relatively uniform deformation under loading, whereas valves incorporating native porcine aortic valve fiber architecture display pronounced spatial heterogeneity, particularly within the leaflet belly region. The fully pressure‐loaded FE results are quantified using maximum in‐plane Green–Lagrange strain (MIPE). The porcine valve (Figure 3c) demonstrates marked heterogeneity in deformation, in contrast to the more homogeneous patterns observed in the pericardial (Figure 3b) and isotropic (Figure 3a) models, especially in the top‐down view. These latter two configurations show comparable deformation magnitudes, with MIPE values of approximately 0.2. Collectively, these findings suggest that aligned collagen fibers can modulate deformation through fiber straightening, highlighting the importance of accurately representing collagen architecture for reliable prediction of BHV fatigue behavior and valve design optimization.

**FIGURE 3:**
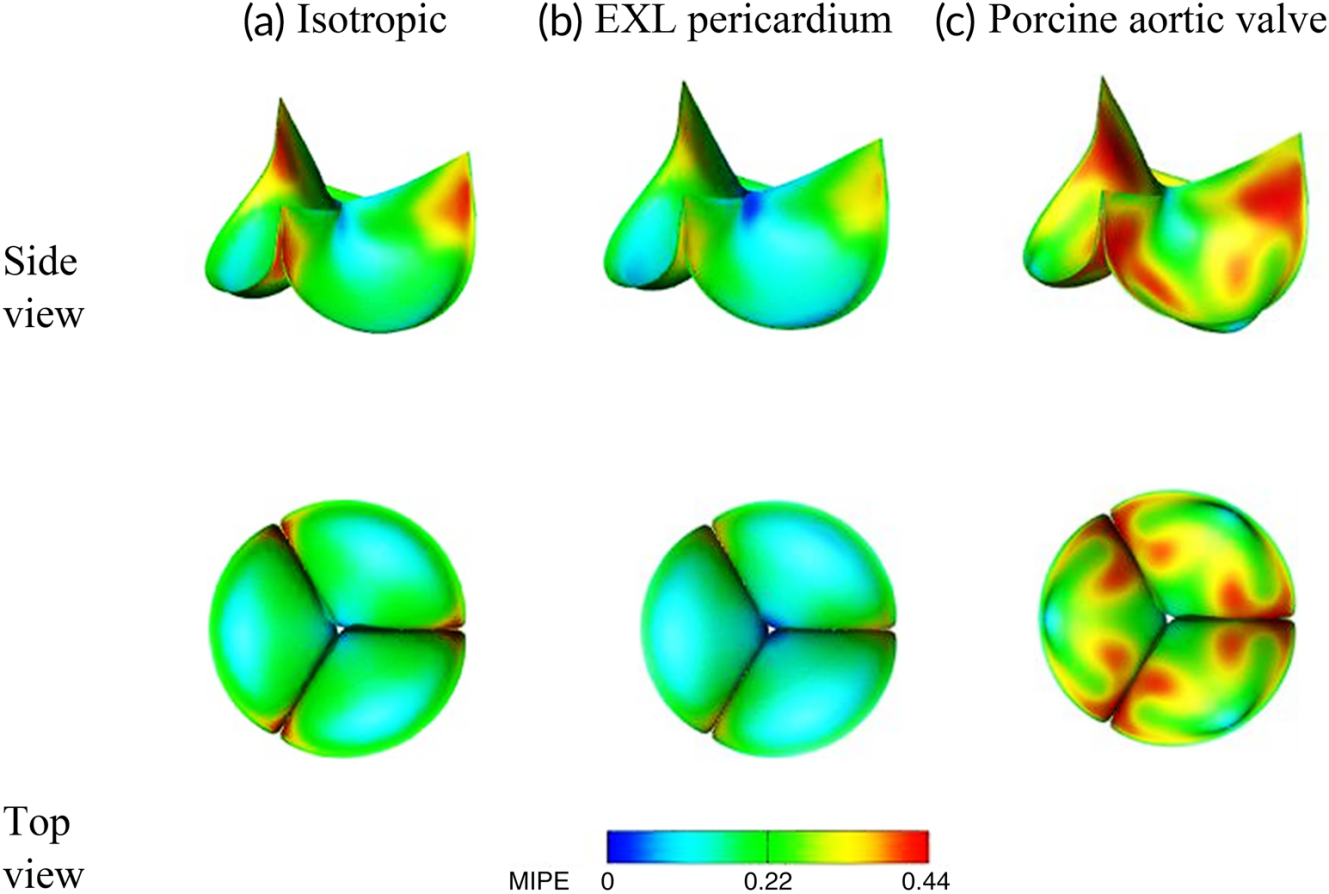
Influence of collagen fiber architecture on leaflet mechanics: Maximum in‐plane Green‐Lagrange strain (MIPE) in the fully pressure‐loaded state. Finite‐element comparison of leaflet deformation patterns across bioprosthetic heart valve (BHV) leaflets incorporating (a) Isotropic fiber architecture, (b) Exogenously crosslinked bovine pericardial fiber architecture, and (c) Native porcine aortic valve fiber architecture, shown from side and top views. Native fiber alignment produces pronounced regional heterogeneity, particularly in the leaflet belly.^26^ (With permission from, 26 License Number 6238580974023).

FE analyses further indicate that stress concentrations preferentially localize within the leaflet belly and near commissural attachments, regions that correspond closely to experimentally observed sites of calcification and structural failure.^36^, ^39^ Multiscale studies demonstrate that calcification initiation is strongly associated with collagen‐rich belly regions and progresses through mineralized fibrillar networks (Figure 4).^39^

**FIGURE 4:**
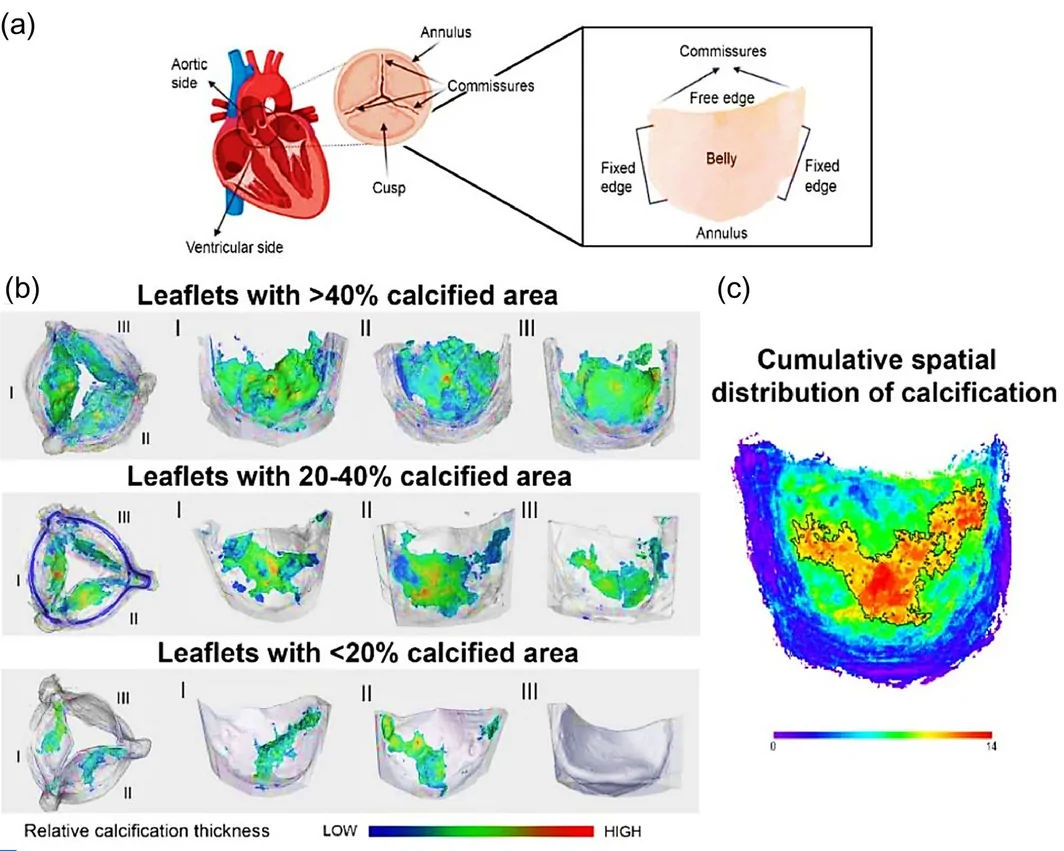
Multiscale characterization of bioprosthetic heart valve (BHV) calcification. Multiscale characterization of calcification initiation and progression in bioprosthetic valve leaflets, integrating imaging and histological analyses.^39^ (With permission under the Creative Commons Attribution 4.0 License).

These observations provide compelling evidence for mechanobiological coupling between cyclic stress exposure, collagen organization, and mineral deposition, underscoring the importance of biomechanical loading history in driving BHV degeneration.

### 2.3 Experimental validation and fluid–structure interaction

Robust validation of FE‐based BHV models necessitates integration with experimental platforms capable of reproducing physiological flow conditions. To this end, high‐fidelity fluid–structure interaction (FSI) models based on the immersed boundary method have been developed to simulate BHV dynamics within pulse‐duplicator systems (Figure 5).^35^ These models employ anatomically realistic geometries and incorporate reduced‐order Windkessel representations to replicate physiological pressure and flow boundary conditions.

**FIGURE 5:**
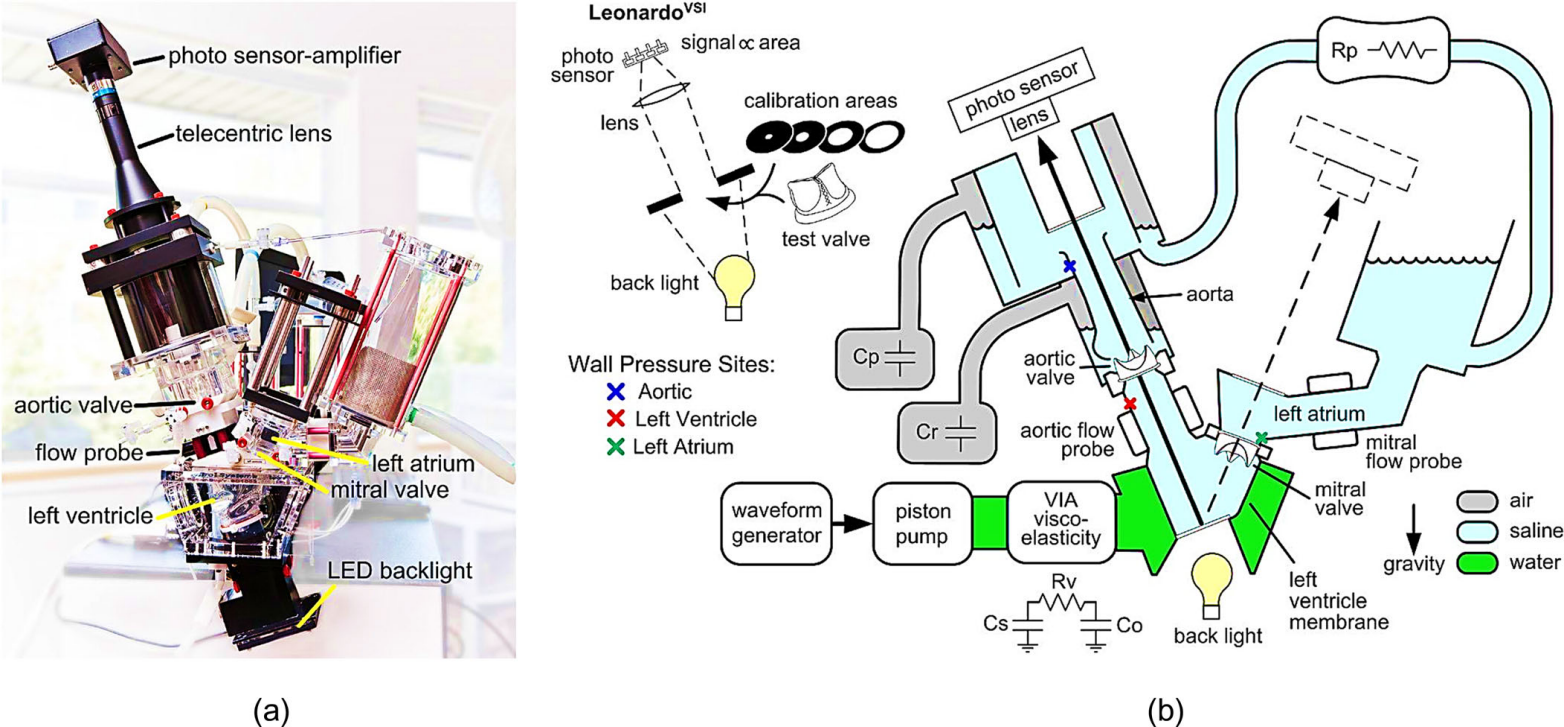
Experimental pulse‐duplicator platform for bioprosthetic heart valve (BHV) validation. Schematic of the experimental pulse‐duplicator system used for validating fluid–structure interaction simulations of BHVs.^35^ (With permission under the Creative Commons Attribution 4.0 License).

Model predictions demonstrate good qualitative agreement with experimental measurements of leaflet kinematics, including sequential leaflet closure and large‐scale flow features, as shown in Figure 6.^35^ Remaining discrepancies between simulations and experiments are attributed to factors such as inter‐leaflet geometric variability, differences in collagen fiber architecture, and unresolved turbulent flow effects at physiological Reynolds numbers on the order of 20,000.^26^, ^40^

**FIGURE 6:**
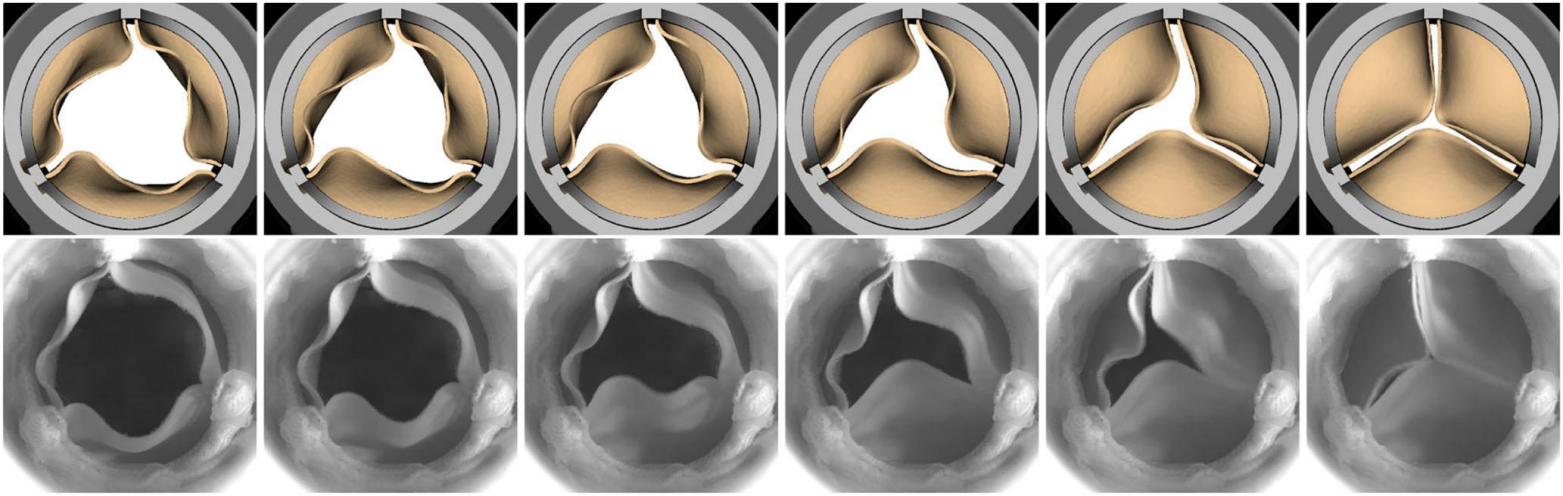
Validation of simulated leaflet kinematics. Comparison between experimentally observed and numerically simulated leaflet kinematics during valve opening and closure.^35^ (With permission under the Creative Commons Attribution 4.0 License).

### 2.4 Clinical translation, computational valve assessment, and relevance to HF

Patient‐specific computational modeling has been successfully extended to transcatheter aortic valve replacement (TAVR), where simulations based on preoperative computed tomography (CT) imaging predict device expansion behavior, anchorage stability, and paravalvular leakage risk (Figure 7).^41^ These models highlight the critical influence of calcific plaque distribution on device deployment and postimplantation mechanics.

**FIGURE 7:**
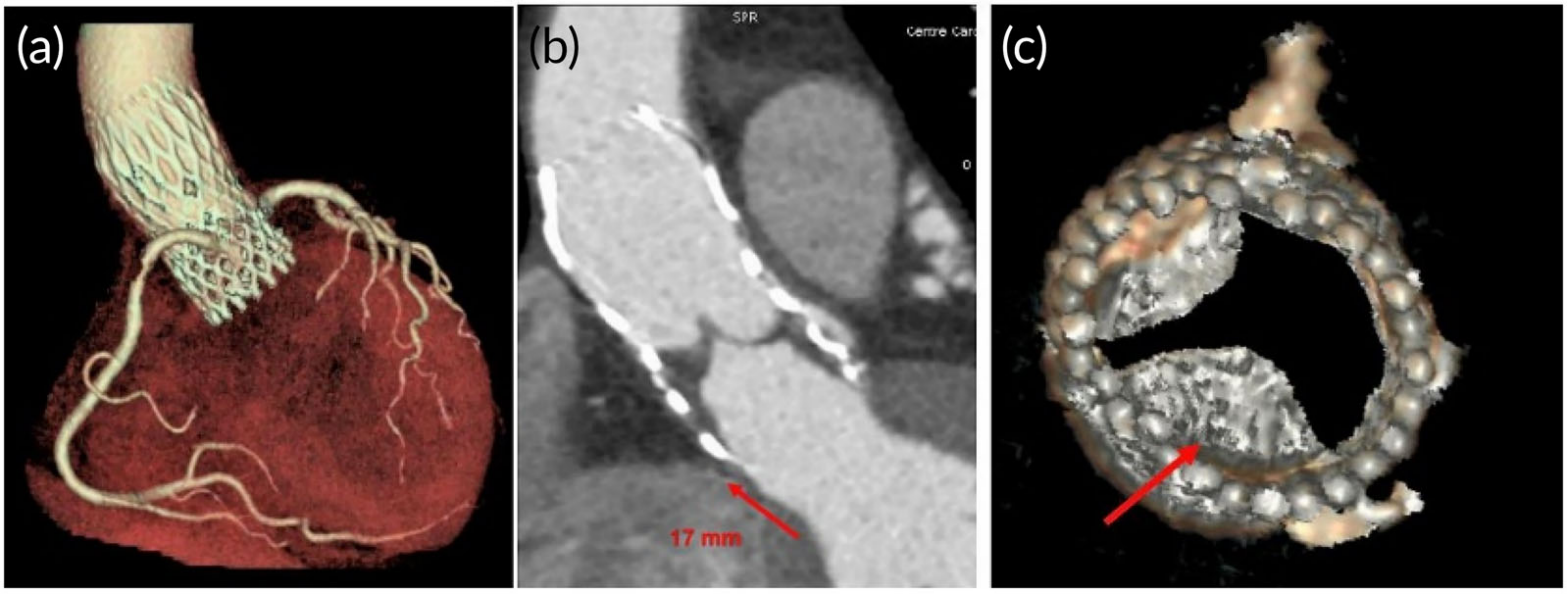
Patient‐specific computational assessment of transcatheter aortic valve replacement (TAVR). TAVR positioning, thrombus development, and patient‐specific biomechanical modeling of the aortic root derived from computed tomography (CT) imaging.^41^ (a) Preoperative CT scan of CoreValve (26 mm). (b) Postoperative CT scan at 1 year shows a device upward shift. (c) Thrombus formation in subvalvular zone (red arrow). No stress map is shown in this figure. (With permission under the Creative Commons Attribution 4.0 License).

In parallel, in silico valve testing frameworks have demonstrated strong correlation with in vitro hydrodynamic performance metrics, including effective orifice area, regurgitant fraction, and transvalvular pressure gradients.^42^ Representative valve geometries, characteristic dimensions, and FE meshes are mentioned in.^42^ Recent computational studies have highlighted advances in multiphysics cardiac modeling, including improved coupling between structural mechanics and hemodynamics, further supporting the role of high‐fidelity simulations in understanding patient‐specific cardiac function and disease progression.^6^, ^35^

## 3 ADDITIVE MANUFACTURING (3D PRINTING) IN CARDIOLOGY

### 3.1 Rationale and evolution of 3D printing in cardiovascular medicine

Additive manufacturing, more commonly known as 3D printing, has emerged as a transformative tool in cardiovascular medicine by enabling the fabrication of patient‐specific anatomical and functional models directly derived from clinical imaging datasets. The growing anatomical complexity of both congenital and acquired heart disease, together with the increasing demand for individualized structural interventions, has driven the widespread adoption of 3D printing for anatomical visualization, procedural planning, medical education, and device evaluation.^43^, ^44^ A broad spectrum of cardiovascular pathologies has been modeled using this approach, including intracardiac defects, vascular malformations, and airway abnormalities, as illustrated in Figure 8.^43^

**FIGURE 8:**
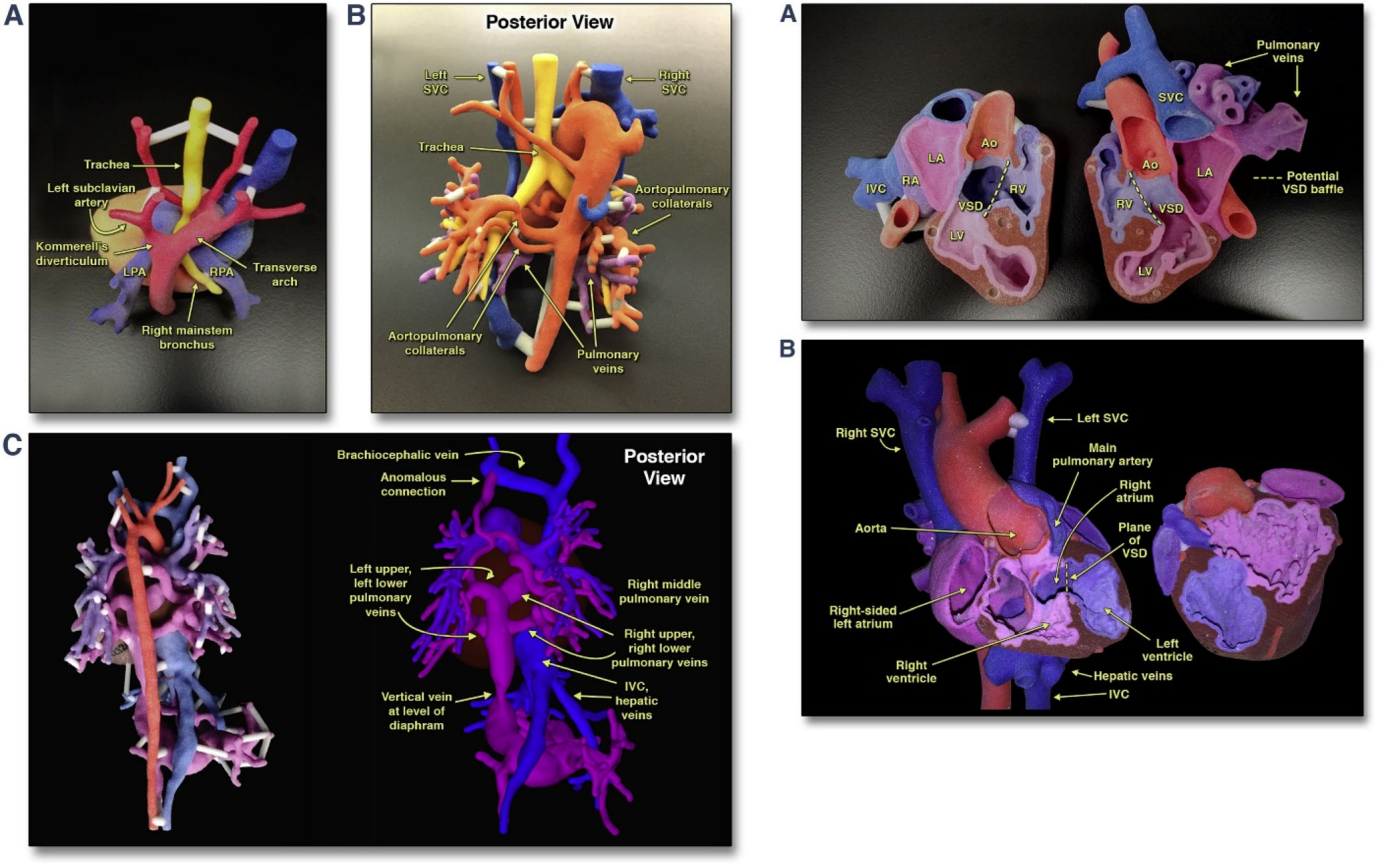
Three‐dimensional (3D) printed models of complex cardiovascular anatomy. 3D printed models of complex intracardiac and extracardiac heart disease across pediatric and adult patients, demonstrating their role in surgical planning, education, and anatomical visualization.^43^ (With permission from, 43 Order No. 6238600310574).

Initial cardiovascular applications of 3D printing focused primarily on static anatomical replication based on CT imaging datasets. With subsequent advances in CMR, three‐dimensional transesophageal echocardiography (3D TEE), and increasingly sophisticated image segmentation methodologies, the scope of 3D printing has expanded to encompass a wider range of structural and functional cardiac pathologies.^44^, ^45^, ^46^, ^47^, ^48^, ^49^, ^50^, ^51^ These developments have positioned 3D printing as a critical translational interface linking medical imaging, computational modeling, and hands‐on clinical decision‐making.

Clinical experience has demonstrated particular value in pediatric and congenital heart disease (CHD), where complex 3D relationships among cardiac chambers, great vessels, and airways are often difficult to fully appreciate using conventional two‐dimensional imaging alone. Patient‐specific printed models have enabled precise preoperative planning in infants with vascular rings, tetralogy of Fallot with pulmonary atresia, total anomalous pulmonary venous return, and other complex intracardiac and extracardiac anomalies. By physically reproducing patient anatomy, these models have facilitated improved surgical strategy selection, enhanced interdisciplinary communication, and more effective education of trainees and caregivers, as shown in Figure 8.

### 3.2 Image acquisition and segmentation for patient‐specific modeling

Image segmentation constitutes the foundational step in the 3D printing workflow, enabling the transformation of volumetric medical imaging datasets into patient‐specific digital anatomical models. Segmentation pipelines typically begin with the export of imaging data in Digital Imaging and Communication in Medicine (DICOM) format, followed by intensity‐based thresholding and iterative manual refinement to accurately isolate target cardiovascular structures.^44^

Historically, segmentation for cardiovascular 3D printing has relied predominantly on CT imaging due to its high spatial resolution and robust depiction of calcified and vascular structures.^52^, ^53^, ^54^, ^55^, ^56^ More recently, CMR imaging has been increasingly employed to reconstruct congenital heart defects and systemic vascular anatomy with high geometric fidelity, particularly in younger patients and those requiring serial imaging without ionizing radiation exposure.^57^, ^58^, ^59^, ^60^, ^61^ In parallel, the feasibility of reconstructing mitral valve leaflets and annular geometry from 3D TEE datasets has been demonstrated by multiple groups.^43^, ^44^, ^45^, ^46^, ^47^, ^48^, ^49^, ^50^ Hybrid segmentation strategies that combine CT‐derived structural detail with echocardiography‐based leaflet morphology have further improved geometric accuracy and functional representation.^49^, ^51^

Once segmentation is completed, the resulting masks are converted into stereolithography (STL) files to enable fabrication. These digital models often undergo additional modification using computer‐aided design (CAD) software to tailor the geometry for specific clinical or experimental objectives, including integration into flow‐loop systems, color‐coding of anatomical regions, or assignment of region‐specific material properties.^49^ The overall workflow from image acquisition to fabrication of a multimaterial patient‐specific model is summarized in Figure 9.

**FIGURE 9:**
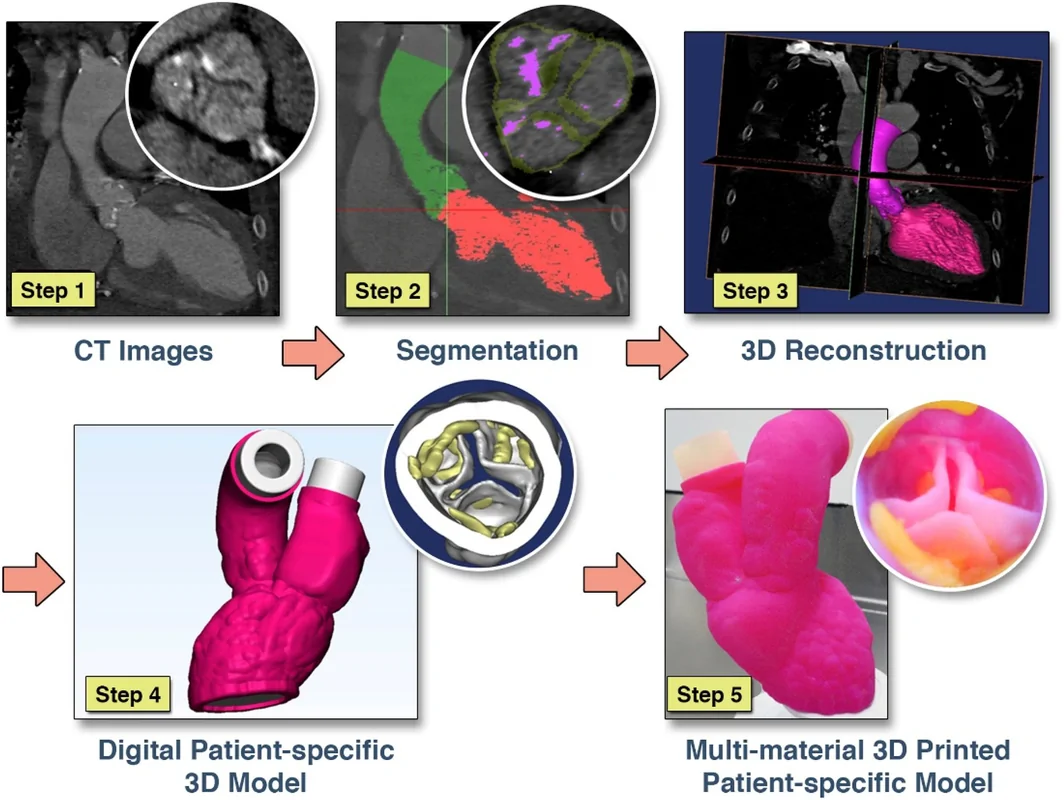
Workflow for patient‐specific three‐dimensional (3D) printing. 3D printed modeling of patient‐specific anatomy illustrating the sequential workflow from computed tomography (CT) image acquisition and segmentation to digital model refinement and multimaterial fabrication.^44^ (With permission from, 44 Order No. 6238610307736).

Cardiovascular 3D printing commonly employs material jetting (PolyJet), SLA, and fused deposition modeling (FDM), each with distinct implications for geometric fidelity, mechanical realism, and reproducibility. PolyJet is particularly suited to multimaterial fabrication because it can combine compliant and rigid regions within a single model, making it useful for patient‐specific cardiovascular phantoms that require both anatomical accuracy and regionally differentiated mechanics. SLA offers high spatial resolution and smooth surface finish, which is advantageous for reproducing fine anatomical detail and thin‐walled structures, although resin selection and postcuring conditions strongly influence the final mechanical response.^62^ FDM is more accessible and cost‐effective, but its output is generally more anisotropic and less anatomically refined because model quality depends on layer height, nozzle temperature, raster orientation, infill density, and cooling conditions.^63^ Across these platforms, key technical parameters that should be reported include imaging source, segmentation workflow, file conversion steps, layer thickness, print orientation, support strategy, curing or postprocessing conditions, and the elastic properties of the selected material. For HF‐related applications, geometric fidelity alone is insufficient; mechanical fidelity must also be characterized under use‐specific loading conditions so that the printed model can be interpreted appropriately as an anatomic, procedural, or biomechanical surrogate.^64^

### 3.3 Educational, communication, and training applications

Patient‐specific 3D‐printed cardiac models have demonstrated substantial value in medical education, surgical rehearsal, and patient‐physician communication. Physical models representing complex CHD, valvular abnormalities, and vascular malformations have been widely used to enhance anatomical understanding among trainees, nurses, and allied healthcare professionals, while also improving procedural confidence and informed consent discussions with patients and families.^65^, ^66^, ^67^

Beyond professional training, 3D‐printed models provide a tangible medium for patient education by enabling visualization of otherwise abstract anatomical and pathophysiological concepts. Multimaterial and multicolored heart models can be selectively simplified or intentionally exaggerated to illustrate disease progression and long‐term hemodynamic consequences of untreated defects. For example, progressive right atrial and right ventricular dilation associated with atrial septal defect (ASD) has been effectively demonstrated using sequential models that depict increasing chamber enlargement over time, as shown in Figure 10.^68^

**FIGURE 10:**
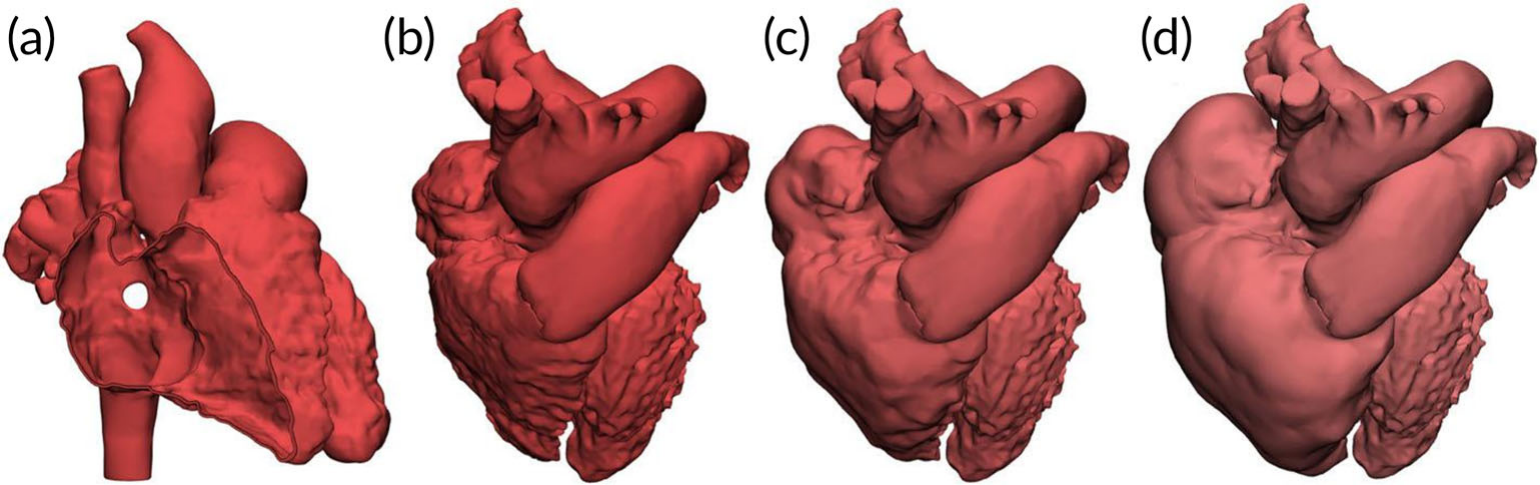
Progressive chamber remodeling in atrial septal defect (ASD). Series of three‐dimensional (3D) printed heart models illustrating progressive right atrial and ventricular dilation resulting from long‐term untreated ASD.^68^ (With permission under the Creative Commons Attribution 4.0 License).

### 3.4 Functional flow models and hemodynamic replication

Beyond static anatomical representation, 3D printing has enabled the development of functional cardiac phantoms capable of replicating patient‐specific hemodynamics when integrated with pulsatile flow‐loop systems. Advances in multimaterial printing allow the simultaneous fabrication of compliant soft tissue regions and rigid calcified components, a feature particularly relevant for modeling aortic stenosis (AS), as demonstrated in Figure 11a.

**FIGURE 11:**
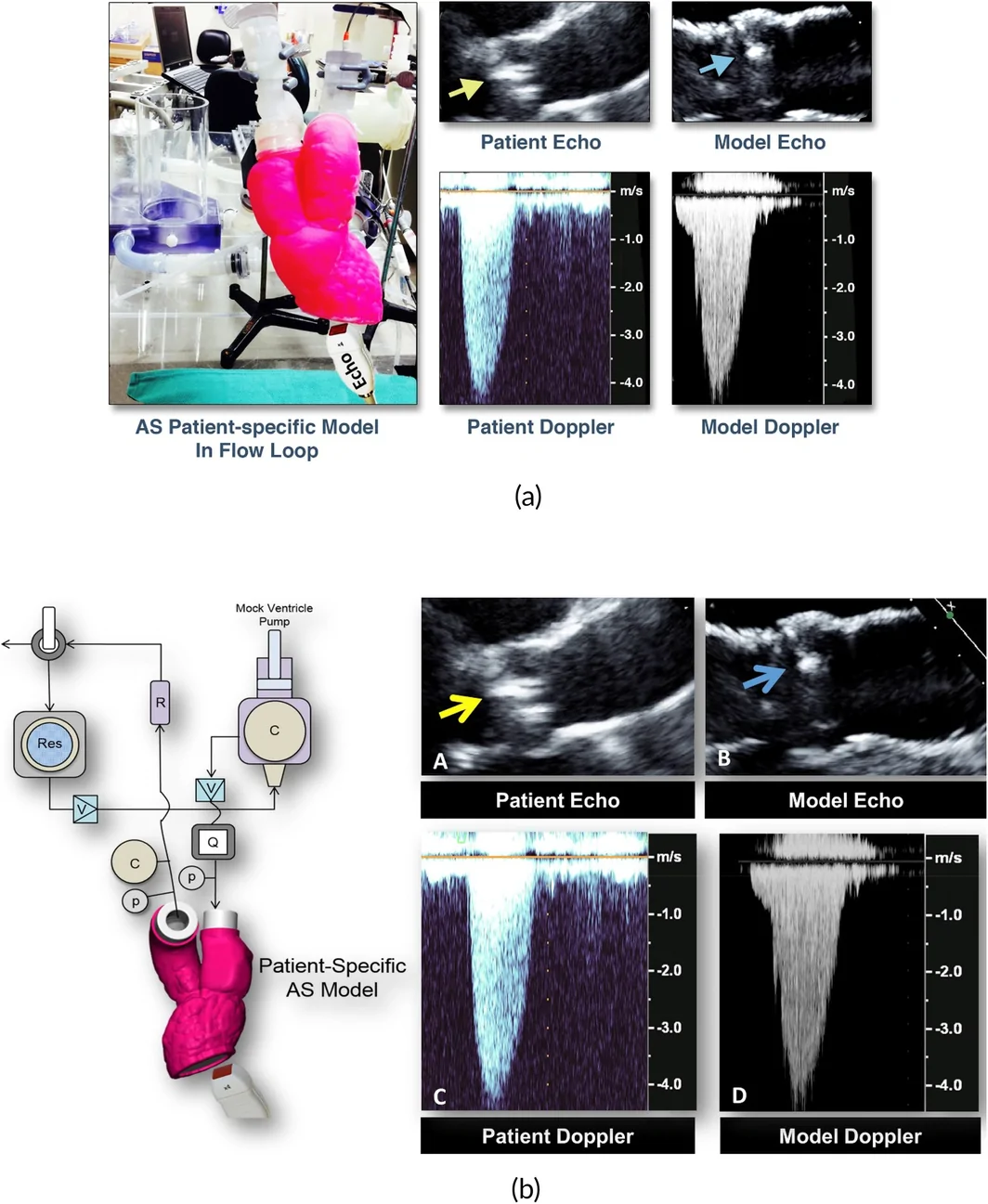
Functional flow modeling of aortic stenosis (AS). (a) Functional modeling of patient‐specific AS using three‐dimensional (3D) printed phantoms coupled with pulsatile flow‐loop systems.^44^ (With permission from, 44 Order No. 6238610307736). (b) Flow‐loop platform used to assess reproducibility of hemodynamics in a 3D printed valve model, with comparison to patient echocardiographic data.^69^ (With permission from, 69 Order No. 6238611159988).

When coupled with physiological flow loops, these patient‐specific phantoms have been shown to reproduce echocardiographic imaging features and hemodynamic profiles characteristic of severe AS. Experimental measurements obtained from such systems demonstrate close agreement with Doppler‐derived transvalvular gradients and effective orifice area calculations, supporting their validity as physiologically meaningful testing platforms.^56^

Maragiannis et al.^56^ further demonstrated the feasibility of constructing multimaterial models from cardiac computed tomography (CCT)‐based datasets that accurately replicated the severity of AS. In their study, flow‐loop measurements obtained from 3D‐printed outflow tract models closely matched patient echocardiographic data, as shown in Figure 11b.

### 3.5 Structural heart interventions and procedural planning

One of the most clinically impactful applications of 3D printing has been in the planning and simulation of catheter‐based structural heart interventions, as illustrated in Figure 12a. Patient‐specific models have been widely used to guide TAVR, atrial septal defect closure, mitral valve repair, and VAD placement by enabling bench‐top device deployment and procedural rehearsal.^44^, ^57^, ^69^, ^70^, ^71^, ^72^

**FIGURE 12:**
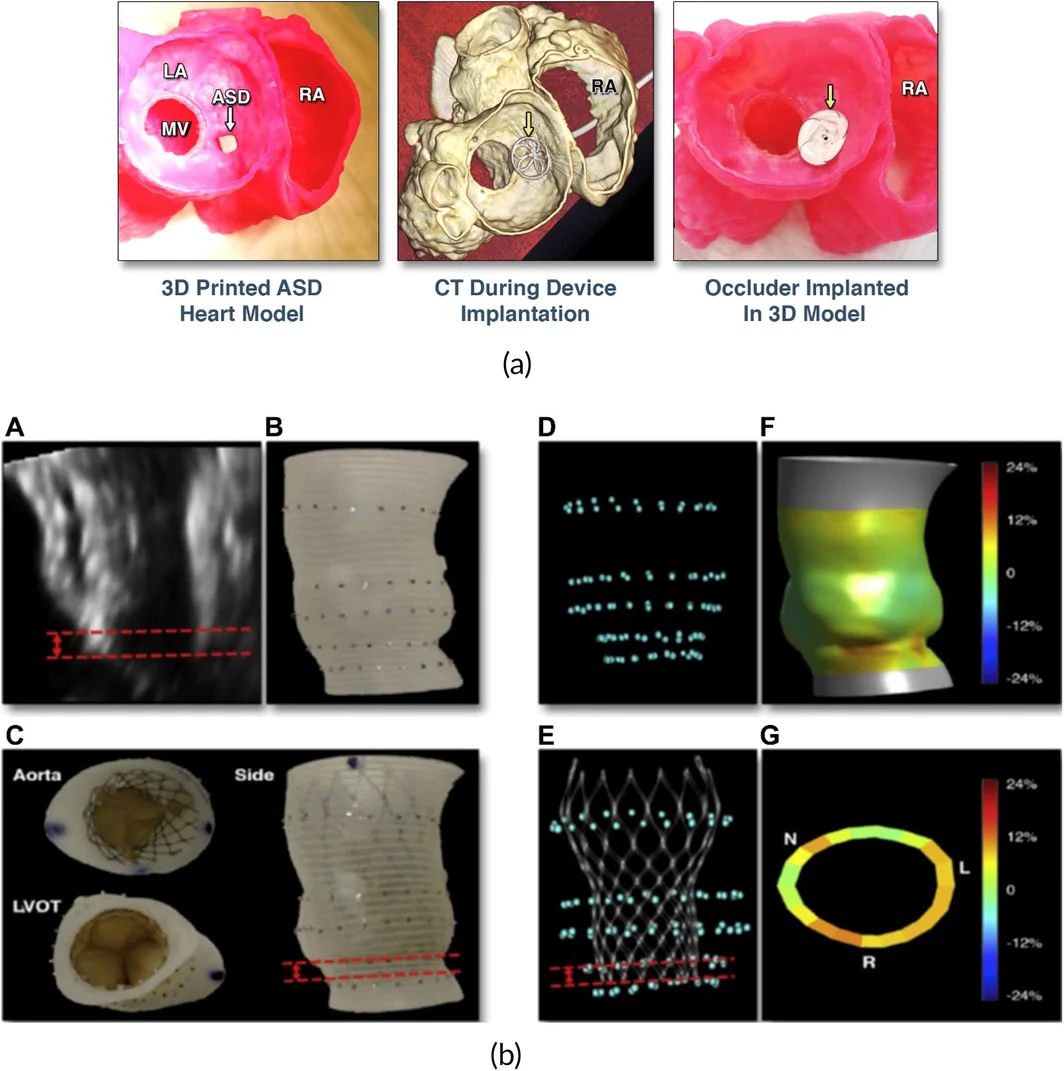
Structural heart intervention planning using three‐dimensional (3D) printing. (a) Preprocedural planning of catheter‐based atrial septal defect closure using a patient‐specific 3D printed model.^44^ (With permission from, 44 Order No. 6238610307736). (b) Transesophageal echocardiographic images and corresponding 3D printed phantom illustrating strain distribution following transcatheter valve implantation.^69^ (With permission from, 69 Order No. (6238611159988).

Such models allow direct visualization of device‐anatomy interaction and provide a controlled environment for assessing procedural feasibility and potential complications. Quantitative prediction of paravalvular leak using tissue‐mimicking aortic root phantoms has been demonstrated by Qian et al.,^72^ who introduced biomechanically informed parameters such as the maximum bulge index to characterize sealing performance, as shown in Figure 12b.^69^

### 3.6 Heart failure and adult congenital heart disease applications

In patients with adult congenital heart disease (ACHD) complicated by HF, 3D printed models have provided critical insight into complex anatomical substrates that are difficult to fully characterize using conventional imaging alone. These include systemic right ventricular anatomy following atrial switch procedures, Fontan physiology, and complex baffle geometries, as illustrated in Figure 13.^73^

**FIGURE 13:**
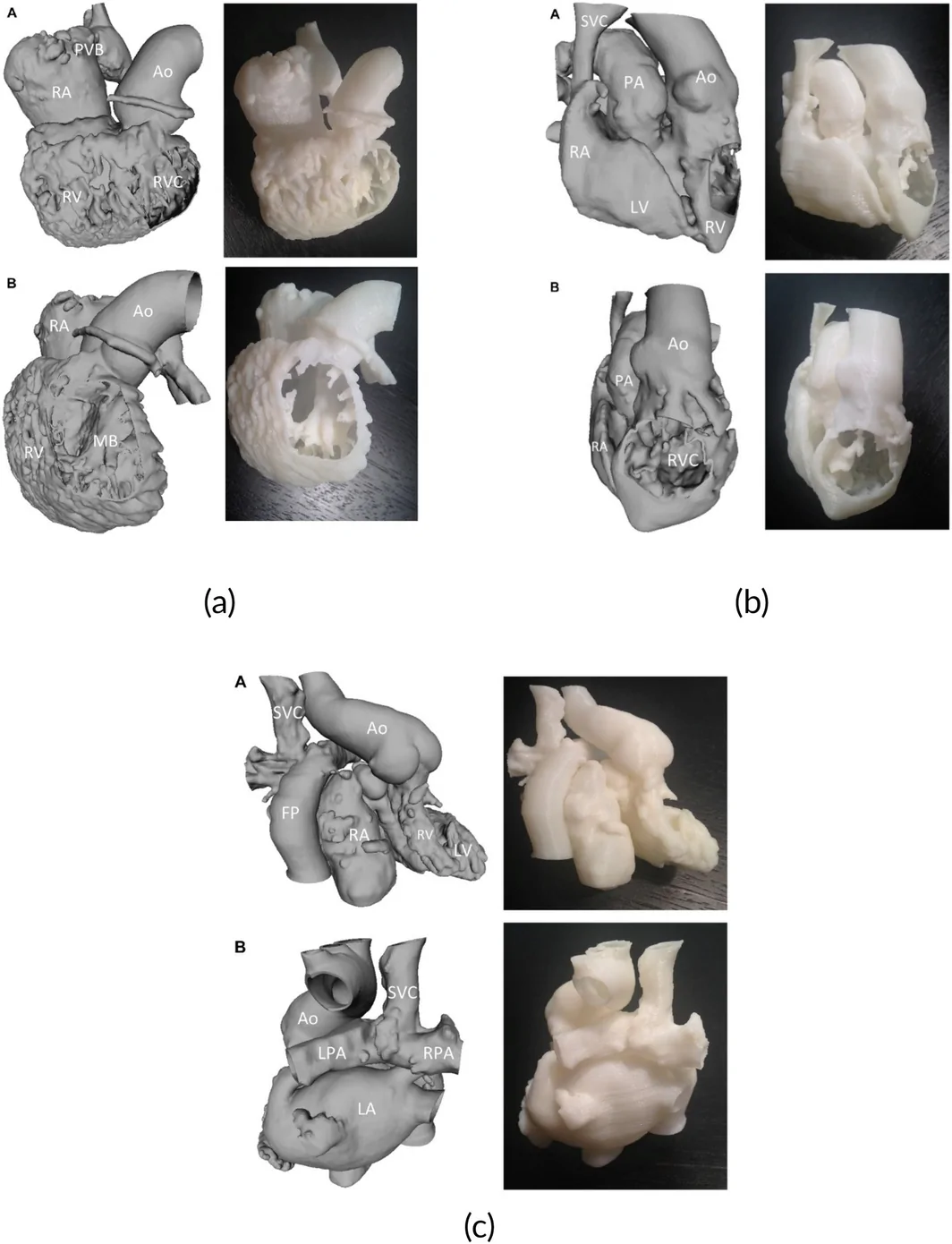
Three‐dimensional (3D) printed models in adult congenital heart disease (ACHD) and heart failure (HF). (a) 3D virtual and printed models illustrating pulmonary venous baffle anatomy following Mustard procedure in a patient with systemic right ventricular failure.^73^ (b) 3D printed model of a patient with a systemic right ventricle highlighting hypertrophied trabeculations and anterior aortic position.^73^ (c) Visualization of Fontan pathway anatomy using a patient‐specific 3D printed model to support planning of advanced HF therapies.^73^ (With permission from, 73 Order No. (6238610967380).

Printed models have been used to visualize hypertrophied and trabeculated systemic right ventricles, anteriorly positioned great vessels, and surgically constructed pathways, thereby supporting planning for advanced HF therapies, including VAD implantation. Collectively, these applications demonstrate the value of 3D printing in bridging anatomical complexity with individualized HF management strategies.

### 3.7 Coronary, valve, and hybrid training models

3D printing has also been applied to coronary artery visualization, catheter‐based training, and hybrid simulation environments. Patient‐derived coronary models enable visualization of stenotic segments and facilitate hemodynamic assessment within flow systems, as shown in.^44^, ^74^, ^75^

Highly engineered training platforms have been developed to simulate TAVR deployment, coronary occlusion risk, and catheter navigation under fluoroscopic and angiographic imaging. Figure 14 illustrates a transapical TAVR simulation using a patient‐specific printed heart model and a J‐valve system (Jiecheng Ltd., Suzhou, China), enabling stepwise procedural rehearsal and device evaluation.^21^

**FIGURE 14:**
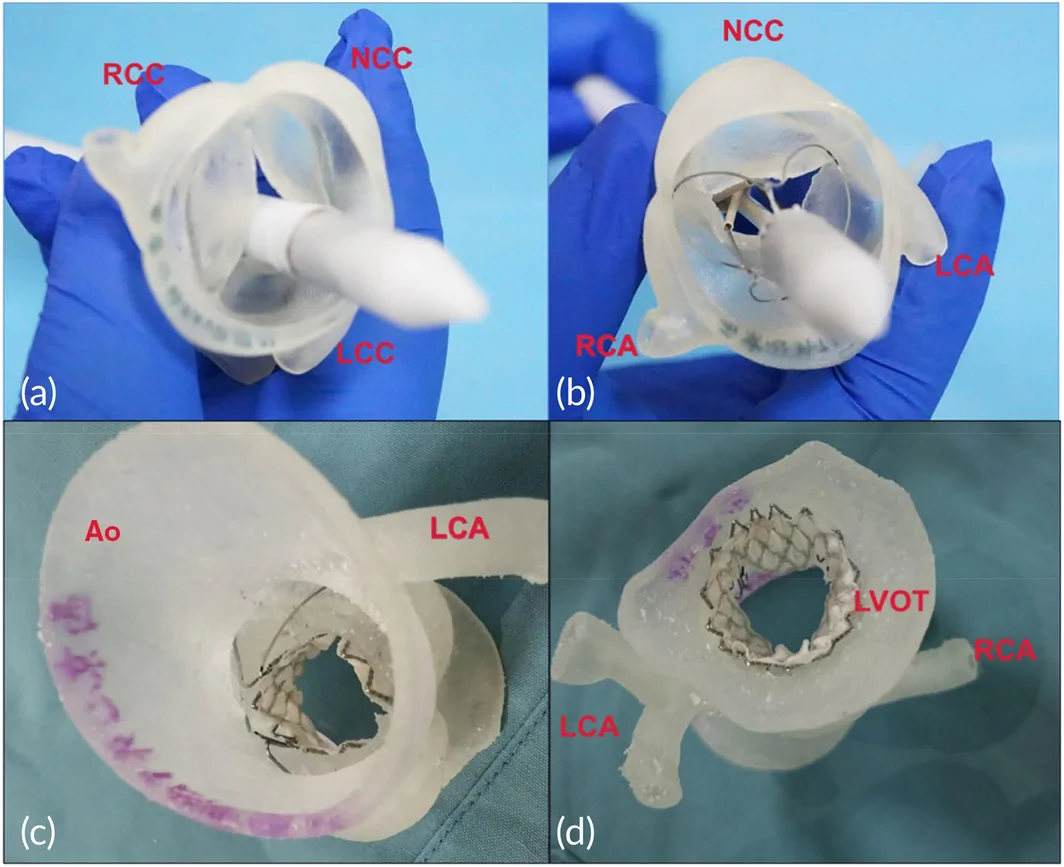
Coronary and hybrid interventional training models. Patient‐specific three‐dimensional (3D) printed heart model used to simulate transapical transcatheter aortic valve replacement and stepwise valve deployment.^21^ (With permission under the Creative Commons Attribution 4.0 License).

Multimaterial 3D phantoms permit benchtop replication of patient hemodynamics for procedural rehearsal and device testing; these models are most valuable when guided by validated FE outputs and when used to change or confirm the planned intervention.

Recent work has further emphasized the integration of data‐driven and physics‐based approaches, demonstrating how hybrid computational frameworks can enhance predictive performance and improve interpretability in cardiac modeling applications, particularly in the context of complex cardiovascular conditions such as HF.^16^, ^18^

We identified no prospective clinical validation studies that directly linked patient‐specific FE descriptors or 3D‐printed phantom testing to hard clinical endpoints (all‐cause mortality or HF hospitalization) in HF populations; existing evidence is limited to retrospective associations, case reports, and bench/phantom validation.

## 4 CLINICAL APPLICATIONS: DIAGNOSIS AND PROGNOSIS USING AI AND ML

### 4.1 Conceptual foundations of AI in cardiovascular medicine

AI broadly refers to computational systems designed to perform tasks that traditionally require human intelligence, including pattern recognition, classification, and decision‐making. ML, a major subset of AI, originated from computer science and statistical learning theory and relies on large datasets to identify complex patterns and relationships without explicit rule‐based programming.^76^, ^77^ A further subset, deep learning (DL), employs artificial neural networks (ANNs) composed of multiple interconnected computational layers inspired by biological neural systems, enabling hierarchical feature extraction and nonlinear decision boundaries, as illustrated in Figure 15.

**FIGURE 15:**
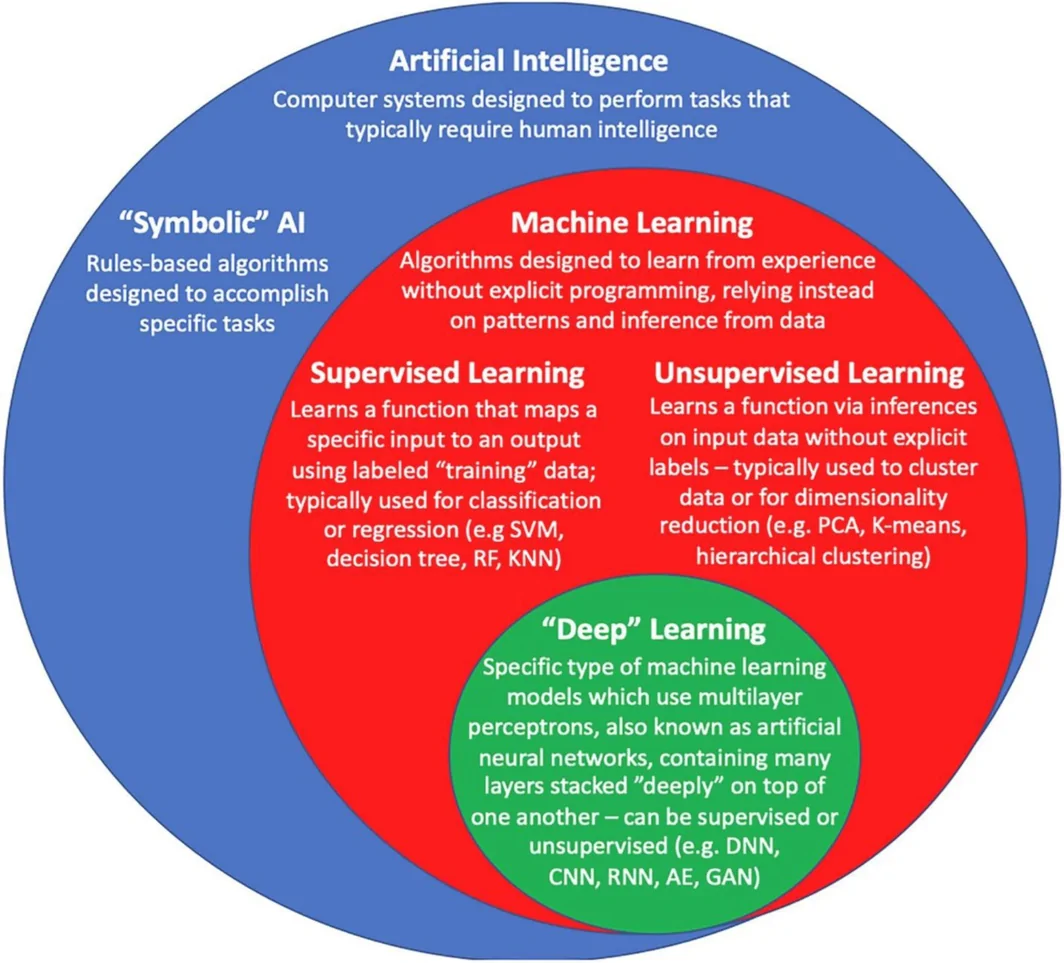
Hierarchy of artificial intelligence (AI) methodologies. Schema illustrating the relationship between AI, machine learning (ML), and deep learning (DL), with representative algorithm classes. SVM = support vector machines; RF = random forests; KNN = k‐nearest neighbor; PCA = principal components analysis; DNN = deep neural network; CNN = convolutional neural network; RNN = recurrent neural network; GAN = generative adversarial network; VAE = variational autoencoder.^76^ (With permission from, 76 Order No. 6238611355483).

In cardiovascular applications, ML workflows typically divide available data into training, validation, and testing subsets. Training datasets are used to estimate model parameters, validation datasets to optimize hyperparameters and prevent overfitting, and independent testing datasets to evaluate generalizability. Strict separation of testing data from model development is essential to avoid information leakage and artificial inflation of predictive performance.^76^

### 4.2 Unmet clinical needs addressed by AI in VHD and HF

AI‐enabled methodologies have been proposed to address several persistent unmet clinical needs in VHD and HF, including:

1. early disease screening,
2. objective quantification of disease severity,
3. individualized risk prediction and outcome stratification, and
4. phenomapping of heterogeneous disease subtypes.^76^

These domains are summarized in the referenced review,^76^ illustrating the breadth of AI applications across diagnostic modalities, disease stages, and clinical workflows.

### 4.3 AI‐based screening and diagnosis

AI has demonstrated significant promise as a scalable screening tool for VHD using nonimaging modalities, including electrocardiography and phonocardiography. An AI‐enabled electronic stethoscope system was developed to detect left‐sided VHD—specifically AS, aortic regurgitation (AR), mitral stenosis (MS), and mitral regurgitation (MR)—using heart sound recordings as input data.^78^ The selection of the study population used to train and validate this model is summarized in Figure 16a, while the signal‐processing pipeline and algorithm development workflow are shown in Figure 16b.

**FIGURE 16:**
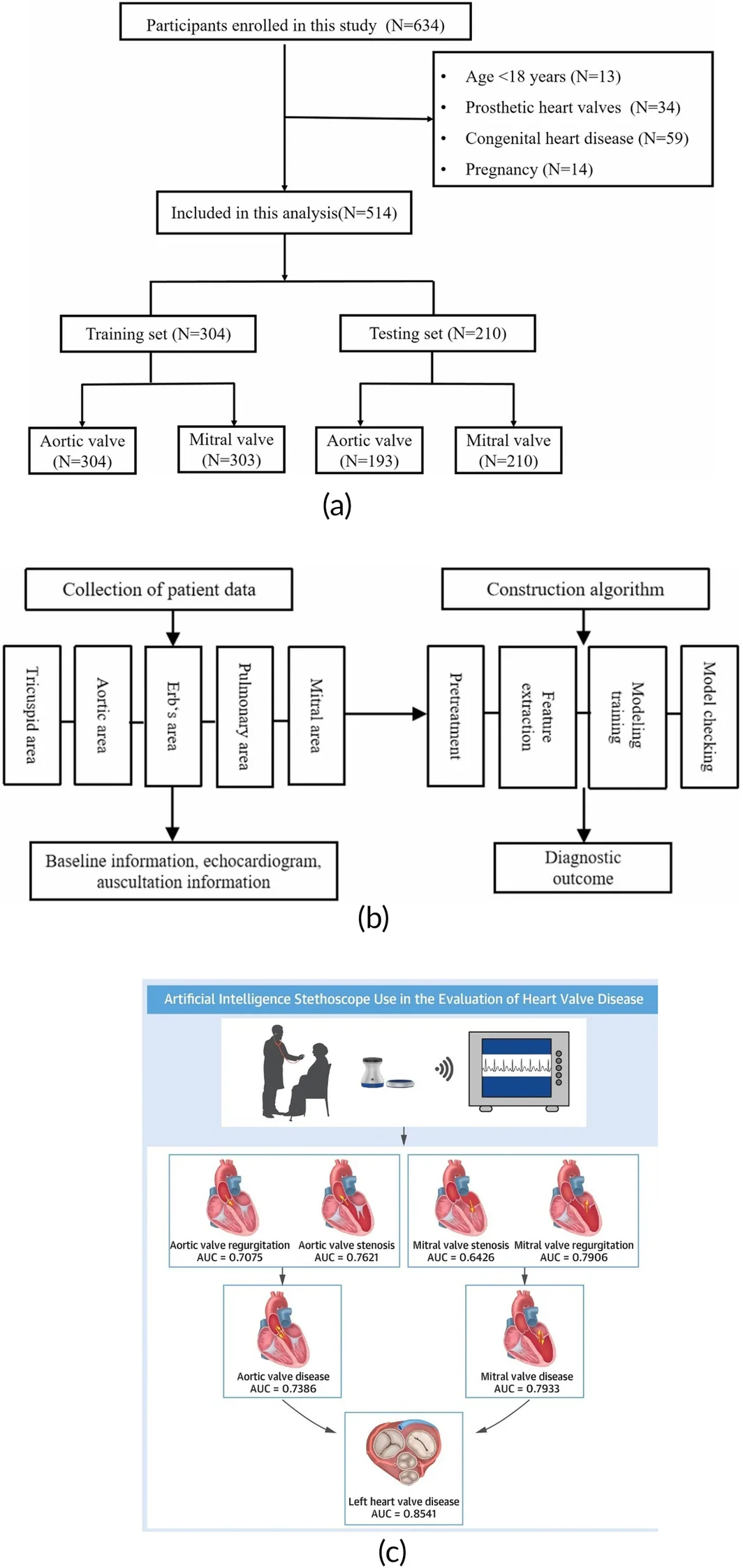
Artificial Intelligence (AI)‐enabled electronic stethoscope for valve disease screening. (a) Flowchart illustrating selection of participants included in the AI‐based electronic stethoscope study.^78^ (b) Overview of heart sound signal acquisition, preprocessing, model development, and diagnostic output generation.^78^ (c) Performance of an AI‐enabled electronic stethoscope in the evaluation of heart valve disease.^78^ (With permission under the Creative Commons Attribution 4.0 License).

In validation testing, the algorithm achieved area under the receiver operating characteristic curve (AUROC) values of 0.76 for AS, 0.71 for AR, 0.64 for MS, 0.79 for MR, and 0.85 for composite left‐sided VHD, supporting its feasibility as a point‐of‐care screening tool, as shown in Figure 16c.^78^

### 4.4 Automated imaging interpretation and anatomical quantification

AI‐driven automation has substantially reduced interobserver variability and improved reproducibility in imaging‐derived cardiovascular measurements. In echocardiography, AI‐based software platforms enable automated quantification of aortic annular dimensions using 3D datasets, allowing both static and dynamic assessment throughout the cardiac cycle.^79^ As illustrated in Figure 17, the automated system reconstructs 3D data in long‐ and short‐axis planes, enabling either single time‐point measurements or continuous annular tracking across systole and diastole.

**FIGURE 17:**
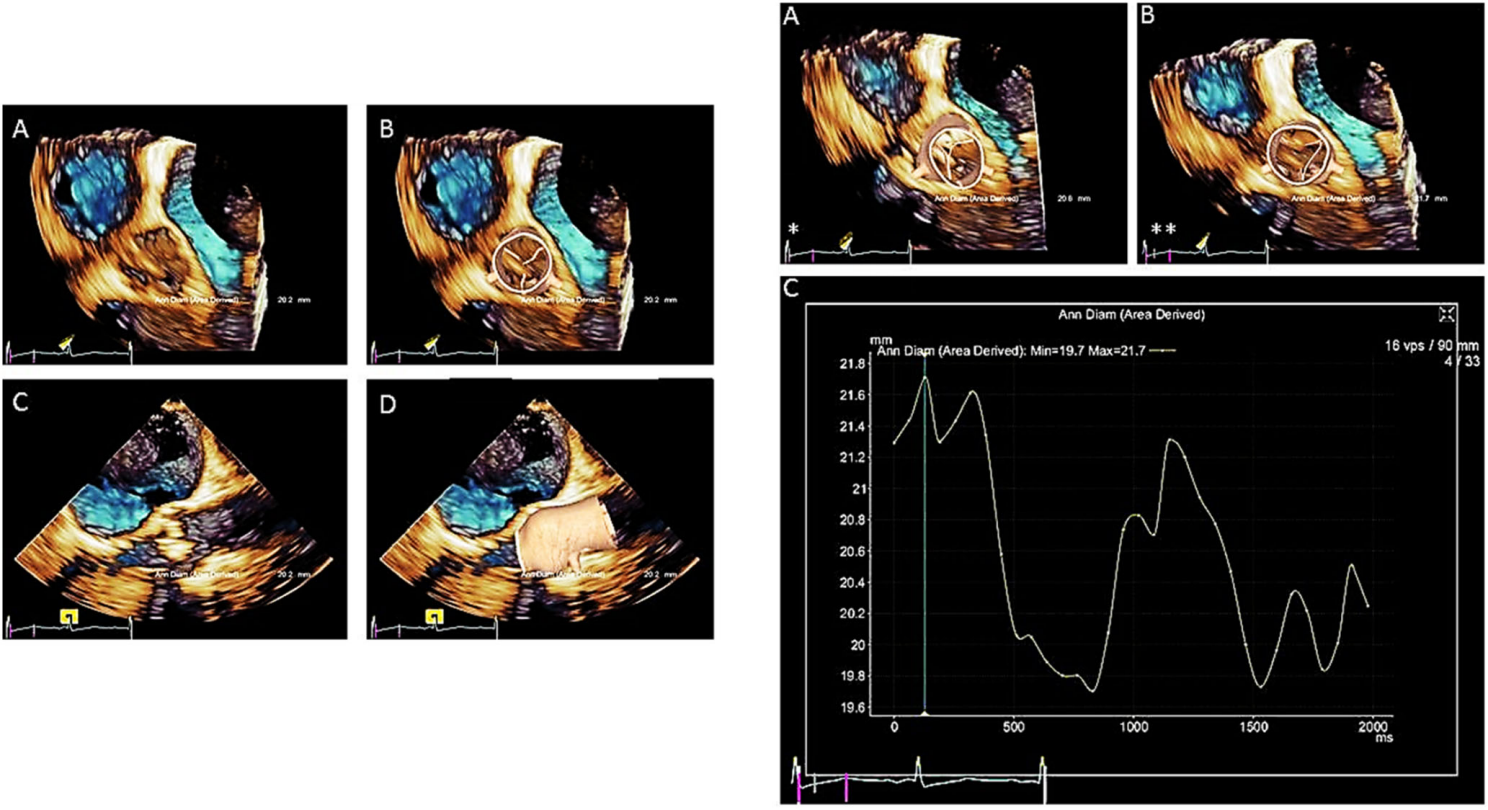
Automated Artificial Intelligence (AI)‐based aortic annulus quantification. Automated aortic annulus measurement using AI‐based software: Static measurements at selected time points and dynamic analysis across the cardiac cycle.^79^ (With permission from, 79 Order No. 6238620392673).

In CT‐based workflows, the 4TAVR system provides a fully automated pipeline encompassing anatomical segmentation, landmark identification, multiplanar reconstruction, measurement extraction, and report generation without user interaction.^80^ The end‐to‐end analysis pipeline is illustrated in Figure 18a, while representative 3D reconstructions and two‐dimensional reference images are shown in Figure 18b.

**FIGURE 18:**
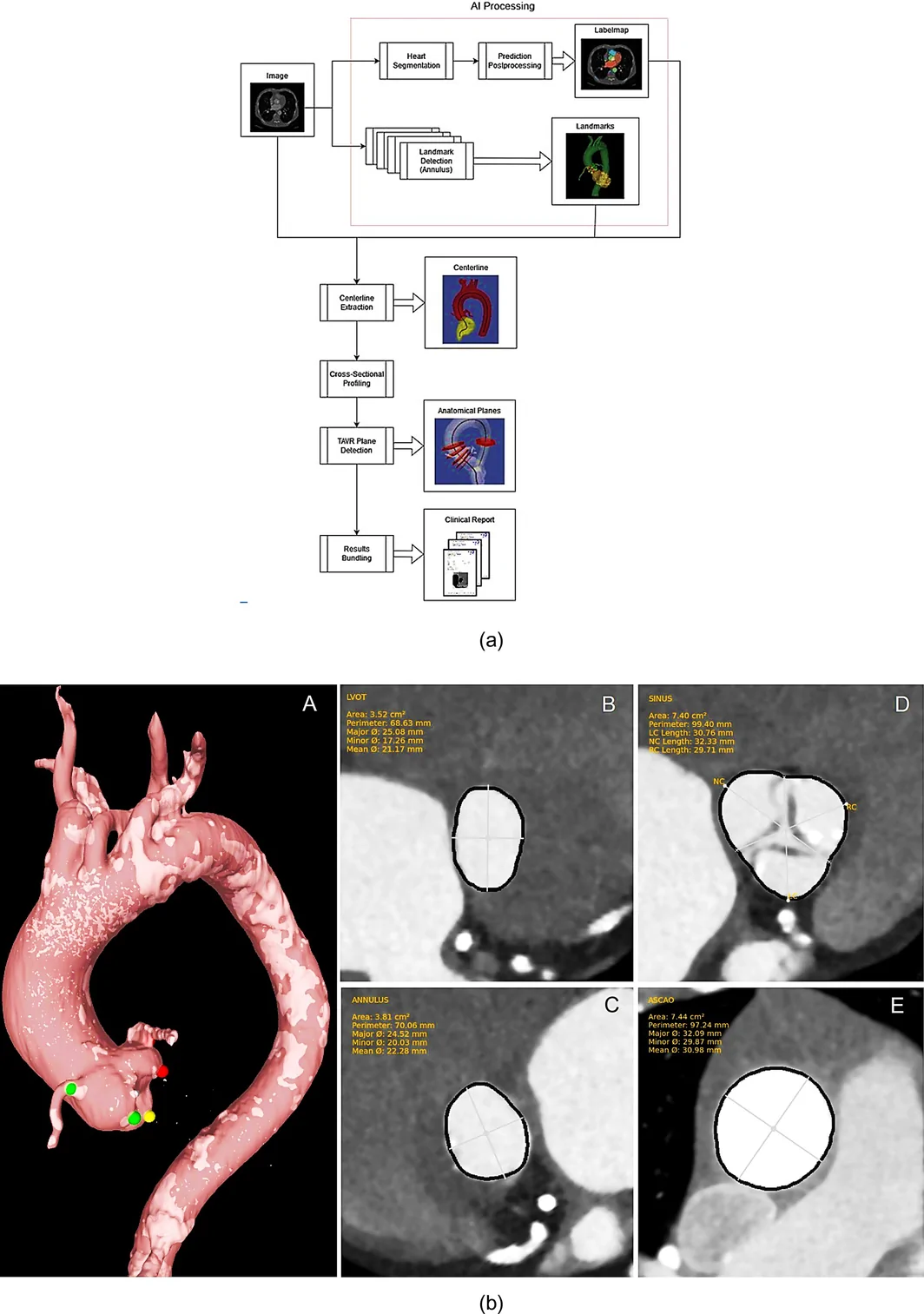
Fully automated computed tomography (CT)‐based transcatheter aortic valve replacement (TAVR) planning pipeline. (a) Automated 4TAVR pipeline including AI processing, centerline extraction, cross‐sectional profiling, TAVR plane detection, and report generation.^80^ (With permission under the Creative Commons Attribution 4.0 License). (b) Visualization of automated anatomical segmentation and quantitative measurements at multiple anatomical levels.^80^ (With permission under the Creative Commons Attribution 4.0 License).

### 4.5 Risk prediction and prognostication

ML algorithms have been extensively applied to predict clinical outcomes following TAVR. Using a large national inpatient dataset, logistic regression (LR), ANNs, naïve Bayes (NB), and random forest (RF) models demonstrated strong discriminative performance for in‐hospital mortality, with LR achieving the highest area under the curve (AUC = 0.92), as shown in Figure 19.^81^

**FIGURE 19:**
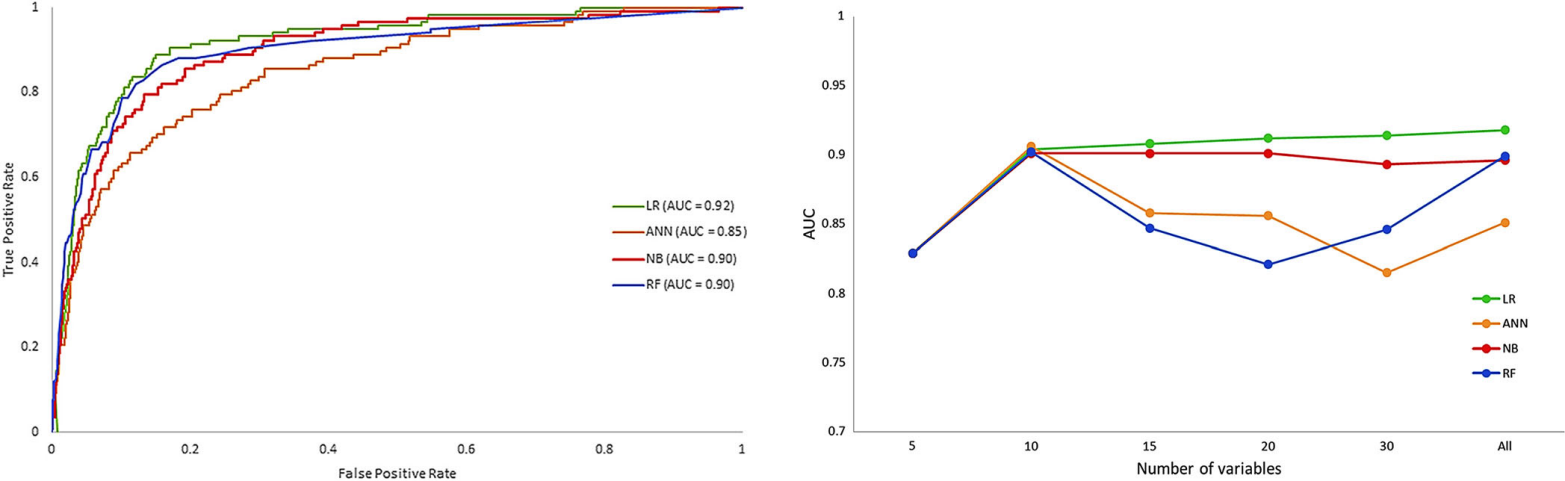
Machine learning prediction of transcatheter aortic valve replacement (TAVR) outcomes. Receiver operating characteristic curves for machine learning (ML) models predicting in‐hospital mortality after TAVR (left) and model performance as a function of variable number (right).^81^ (With permission from, 81 Order No. 6238620175414).

These findings highlight the potential for AI‐based risk prediction tools to outperform conventional scoring systems and to support individualized patient selection, procedural planning, and counseling.^81^

### 4.6 Natural language processing and longitudinal disease surveillance

Natural language processing (NLP), when combined with rule‐based engines, has enabled large‐scale identification and longitudinal surveillance of VHD using both structured and unstructured echocardiographic report data.^82^ Significant discrepancies were identified between quantitative measurements—such as aortic valve area—and narrative report conclusions, underscoring the value of AI‐assisted reconciliation of imaging data.

### 4.7 AI analysis of device‐related and acoustic signals

ML techniques have also been applied to the analysis of acoustic signals generated by left ventricular assist devices (LVADs) to detect complications such as significant AR.^83^ Feature extraction combined with ensemble classification methods identified harmonic amplitude and rotational speed metrics as key predictors of device‐related pathology. Representative signal‐processing workflows and feature distributions are shown in Figure 20.

**FIGURE 20:**
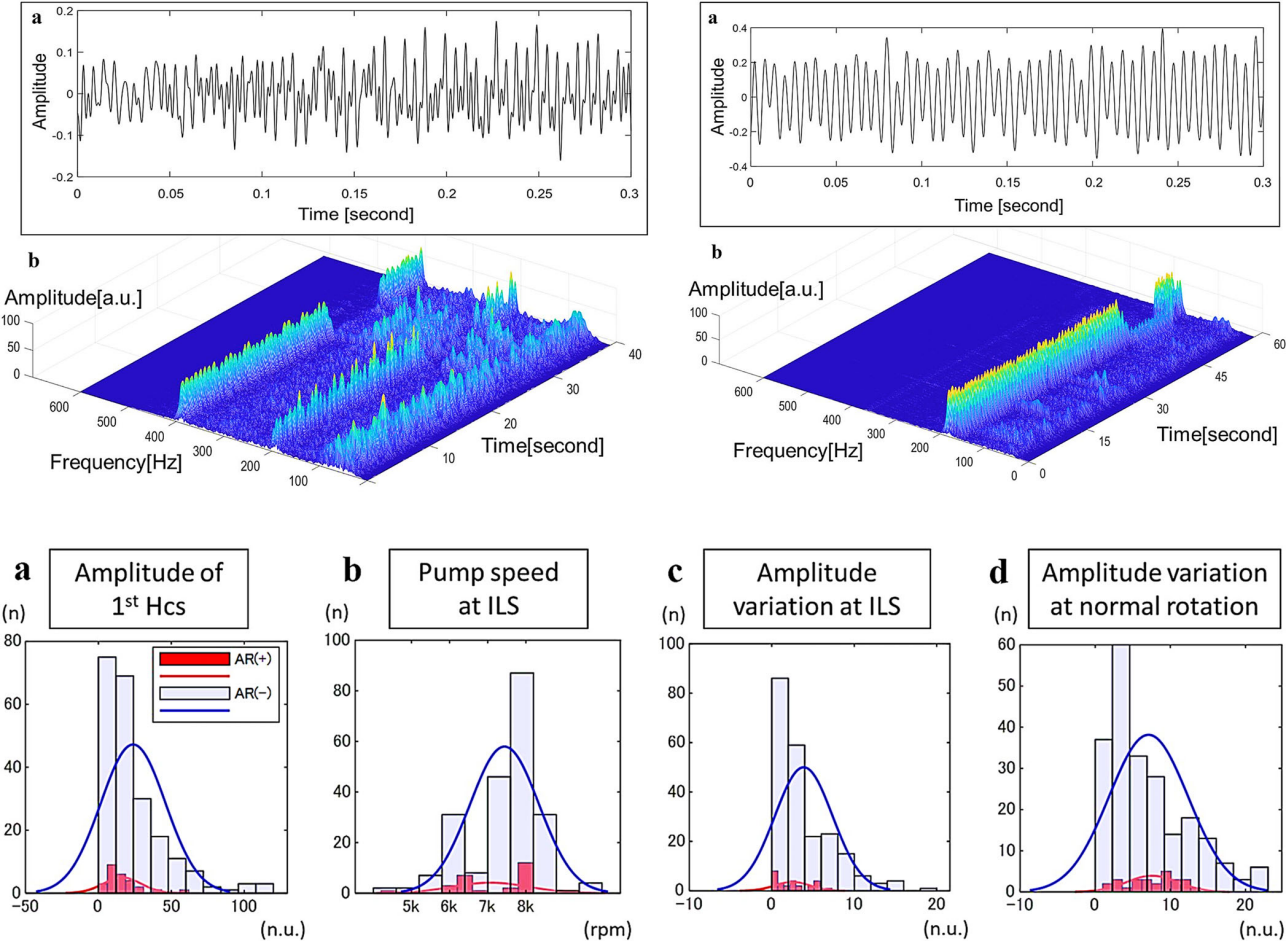
Artificial Intelligence (AI) analysis of left ventricular assist device (LVAD) acoustic signals. LVAD sound signal processing pipeline and distribution of extracted acoustic features associated with device‐related complications.^83^ (With permission under the Creative Commons Attribution 4.0 License).

These approaches demonstrate the expanding role of AI in continuous, noninvasive monitoring of advanced HF therapies.

AI streamlines segmentation, accelerates surrogate modeling, and enables phenomapping from multimodal data—but clinical deployment requires transparent models, external validation, and integration with mechanistic outputs for interpretability.

## 5 EVIDENCE MATURITY AND TRANSLATIONAL READINESS

The translational maturity of FE modeling, AI, and 3D‐printed cardiac phantoms remains heterogeneous. FE approaches are predominantly validated in retrospective or simulation‐based studies, with limited prospective clinical validation. 3D printed phantoms are widely used for in vitro testing and procedural rehearsal; however, direct evidence linking their use to improved patient outcomes remains sparse. In contrast, AI‐based approaches have achieved broader clinical integration, particularly in imaging and risk stratification, although many models rely on retrospective datasets and lack robust external validation. Importantly, differences in study design—including retrospective analyses, preclinical experimentation, and early‐phase clinical studies—complicate direct comparison of predictive performance and reproducibility. Establishing standardized validation pipelines, including prospective trials and multi‐center reproducibility assessments, will be essential for translating these approaches into routine HF care. Direct quantitative comparison of predictive performance across FE models, 3D‐printed phantoms, and AI systems is not currently appropriate because these studies differ substantially in endpoints, datasets, validation standards, and intended clinical use. Accordingly, the comparison below emphasizes evidence type, validation level, and translational readiness rather than pooled accuracy metrics (Table 2).

**TABLE 2:** Translational readiness of finite‐element modeling, three‐dimensional printing, and artificial intelligence in heart failure.

Approach	Evidence type	Validation level	Clinical readiness
FE modeling	Simulation/retrospective	Limited	Low–moderate
3D printing	Bench/in vitro	Minimal	Low
AI	Retrospective + some clinical	Moderate	Moderate

## 6 FUTURE DIRECTIONS AND CONCLUSIONS

The integration of FE modeling, ML, and patient‐specific 3D platforms offers a powerful framework to interrogate core HF mechanisms across scales. Computational models enable quantification of ventricular stress–strain distributions associated with remodeling and fibrosis, while data‐driven approaches can identify phenotypic patterns such as HFpEF heterogeneity and dyssynchrony‐related dysfunction. Similarly, 3D‐printed cardiac phantoms and in vitro systems provide controlled environments to evaluate ventricular–vascular interactions and device–tissue coupling under physiologically relevant conditions. Anchoring these approaches within established HF pathophysiology strengthens mechanistic interpretation and improves their relevance for diagnosis, risk stratification, and therapeutic planning.

### 6.1 Limitations and future directions

Despite substantial progress in data‐driven phenotyping, translation of cardiac mechanics into clinical practice remains limited by nonstandardized definitions, heterogeneous modeling assumptions, and incomplete validation. FE‐derived descriptors are sensitive to imaging quality, segmentation, constitutive modeling, boundary conditions, and mesh resolution, while experimental and phantom studies often lack harmonized reporting. These factors currently impede reproducibility and multicenter comparison.

To address this gap, drawing on prior FE, imaging, and phantom studies,^6^, ^26^, ^44^, ^84^, ^85^, ^86^ we propose a concise set of candidate mechanical descriptors and minimum reporting elements (Table 3). These metrics are proposed as harmonized descriptors to support consistent mechanistic reporting and facilitate future validation.

**TABLE 3:** Candidate mechanical descriptors for mechanistic phenotyping in heart failure.

Descriptor	Literature prevalence (selected refs)	Typical reproducibility /measurement error (reported)	Minimal validation pathway
Peak regional von Mises stress	Reported in patient‐specific FE studies and FSI valve models	Published studies report variable sensitivity to boundary conditions and constitutive assumptions; quantitative uncertainty is inconsistently documented.	Bench (material calibration) → phantom (flow loop comparison of pressure/gradient) → retrospective association with remodeling markers → prospective study with remodeling/HF endpoints.
End‐systolic principal strain	Reported across FE and imaging studies; imaging strain reproducibility established for CMR FT and speckle (~4%–8% depending on vendor).	For imaging: inter‐scan reproducibility 4%–8% (CMR feature‐tracking); for FE: mesh/BC dependent—sensitivity analysis required.	Imaging/FE cross‐validation → phantom test of deformation → retrospective correlation with outcomes → prospective validation.
Strain heterogeneity index (SHI)	Emerging metric in FE and imaging comparisons; referenced in regional remodeling studies.	Not widely reported; depends on segment number and segmentation method.	Standardize segmentation → report SHI reproducibility across readers → retrospective association → prospective study.
Transmural strain gradient	Described in FE and CMR tagging studies as a marker of subendocardial dysfunction.	CMR tagging reproducibility moderate; few multicenter data.	Phantom/ex vivo model validation → retrospective imaging correlation with ischemia/fibrosis → prospective trial for outcome prediction.
Myocardial work density (pressure‐strain loop area)	Increasingly reported as imaging surrogate (pressure‐strain loop) and in FE loops.	Pressure approximation introduces error; reproducibility varies with BP measurement method.	Standardize BP acquisition → bench validation → retrospective association with exercise capacity/ HF admissions → prospective endpoint trial.
Annular displacement/tethering index	Widely reported for functional MR; imaging measures reproducible across modalities with standardized landmarks.	Reproducibility moderate when anatomical landmarks standardized.	Imaging/FE cross‐validation → bench models for leaflet geometry → retrospective outcome associations (repair durability) → prospective registry.
Peak leaflet stress	Reported in valve FE/FSI studies and bench tests; limited clinical correlation.	Strong dependence on leaflet material model; reproducibility not established across teams.	Bench tensile/FSI validation → printed leaflet fatigue testing → targeted clinical correlation with device anchorage/failure → prospective surveillance.
Regional curvature/geometry index	Used in stress concentration analyses and device‐tissue interaction studies.	Depends on smoothing and scale; reproducible if surface parameterization standardized.	Geometry parameter standardization → bench mapping to stress → retrospective correlation with degeneration → prospective testing in device cohorts.

Table 3 summarizes candidate descriptors reported across FE, imaging, and experimental studies and should be interpreted as a research‐stage framework rather than validated clinical biomarkers. Reproducibility and sensitivity to modeling assumptions are variably reported in the literature and should be explicitly addressed in future studies.

Future work should prioritize reproducible, integrated workflows linking imaging, FE modeling, AI analysis, and experimental validation. Such workflows may support the development of patient‐specific digital‐physical twins for hypothesis testing, therapeutic planning, and risk stratification. Prospective, multicenter studies will be essential to establish reproducibility, clinical relevance, and regulatory confidence. Parallel advances in bioprinting, materials science, and AI‐based surrogate modeling may further enhance physiologic fidelity while reducing computational burden. Ethical considerations, including data governance, transparency, and algorithmic bias, must be addressed to ensure responsible clinical adoption.

Taken together, recent studies indicate a transition from isolated modeling or fabrication approaches toward integrated translational platforms in which 3D printing, FE modeling, and machine‐learning‐based phenotyping are jointly optimized. This convergence is particularly relevant for myocardial fibrosis, ventricular remodeling, and personalized HF management, where patient‐specific constructs, computational simulation, and digital‐twin frameworks may enhance risk stratification, procedural planning, and prediction of therapeutic response.^87^, ^88^, ^89^, ^90^, ^91^, ^92^, ^93^, ^94^, ^95^, ^96^, ^97^, ^98^, ^99^ Moreover, emerging work extending these approaches to electrophysiological modeling, vascular remodeling, and device‐based interventions highlights their broader potential for integrated cardiovascular decision support.^100^, ^101^, ^102^, ^103^, ^104^, ^105^

### 6.2 Conclusions

HF is a mechanically complex syndrome that cannot be fully characterized by conventional clinical metrics alone. FE modeling, additive manufacturing, and AI offer complementary tools to quantify cardiac mechanics, experimentally validate computational predictions, and extract clinically meaningful patterns from high‐dimensional data. This review highlights how their integration provides a coherent framework for mechanistic phenotyping and precision HF research. With standardized reporting, rigorous validation, and interdisciplinary collaboration, integrated digital‐physical modeling approaches have the potential to advance HF management toward more predictive, personalized, and mechanistically grounded care. At present, these technologies are best viewed not as standalone clinical tools, but as complementary components of a developing translational ecosystem, where their greatest impact will depend on rigorous validation, integration, and HF‐specific clinical targeting.

## AUTHOR CONTRIBUTIONS

**Quazi Md. Akbar Shah:** Project administration; resources. **Quazi Md Zobaer Shah:** Conceptualization; visualization; writing – original draft; investigation; project administration; writing – review and editing; methodology; supervision; software. **Quazi Noor E Sabrina:** Project administration; resources; investigation; writing – review and editing; supervision; visualization. **Md Mahabub Hasan Mousum:** Resources; project administration; investigation. **Md. Moyeen Uddin Chisty:** Resources; project administration; investigation. **Quazi Noor E Sohela:** Validation; methodology; visualization; investigation.

## FUNDING INFORMATION

This research received no external funding.

## CONFLICT OF INTEREST STATEMENT

The views expressed are those of the authors in their personal academic and research capacity and do not represent the official position, policy, or endorsement of any affiliated institution.

## Data Availability

This article is a narrative review of previously published studies. No new experimental or clinical data were generated, and no statistical analyses were performed by the authors. Figures and tables are intended to schematically summarize concepts, workflows, and representative findings reported in the cited literature. Sample sizes, statistical methods, and data visualization practices are reported in the original sources.

## References

[1] Shahim B , Kapelios CJ , Savarese G , Lund LH . Global public health burden of heart failure: an updated review. Card Fail Rev. 2023;9:e11. doi:10.15420/cfr.2023.05 PMC10398425 37547123

[2] Tsao CW , Aday AW , Almarzooq ZI , et al. Heart disease and stroke statistics‐2023 update: a report from the American Heart Association. Circulation. 2023;147(8):e93‐e621. doi:10.1161/CIR.0000000000001123 PMC12135016 36695182

[3] Pfeffer MA , Shah AM , Borlaug BA . Heart failure with preserved ejection fraction in perspective. Circ Res. 2019;124(11):1598‐1617. doi:10.1161/CIRCRESAHA.119.313572 PMC6534165 31120821

[4] Cho S , Rhee S , Madl CM , et al. Selective inhibition of stromal mechanosensing suppresses cardiac fibrosis. Nature. 2025;642(8068):766‐775. doi:10.1038/s41586-025-08945-9 PMC12176515 40307543

[5] Kim JH , Kiernan MS . Predictors of left ventricular reverse remodeling in heart failure with reduced ejection fraction. Curr Heart Fail Rep. 2025;22(1):39. doi:10.1007/s11897-025-00719-8 41236670

[6] Niederer SA , Lumens J , Trayanova NA . Computational models in cardiology. Nat Rev Cardiol. 2019;16(2):100‐111. doi:10.1038/s41569-018-0104-y PMC6556062 30361497

[7] Palit A , Bhudia SK , Arvanitis TN , Turley GA , Williams MA . Computational modelling of left‐ventricular diastolic mechanics: effect of fibre orientation and right‐ventricle topology. J Biomech. 2015;48(4):604‐612. doi:10.1016/j.jbiomech.2014.12.054 25596634

[8] Otto CM , Nishimura RA , Bonow RO , et al. 2020 ACC/AHA guideline for the management of patients with valvular heart disease: executive summary: a report of the American College of Cardiology/American Heart Association joint committee on clinical practice guidelines. Circulation. 2021;143(5):e35‐e71. doi:10.1161/CIR.0000000000000932 33332149

[9] Sengupta PP , Kulkarni H , Narula J . Prediction of abnormal myocardial relaxation from signal processed surface ECG. J Am Coll Cardiol. 2018;71(15):1650‐1660. doi:10.1016/j.jacc.2018.02.024 29650121

[10] Corral‐Acero J , Margara F , Marciniak M , et al. The digital twin to enable the vision of precision cardiology. Eur Heart J. 2020;41(48):4556‐4564. doi:10.1093/eurheartj/ehaa159 PMC7774470 32128588

[11] Ravi SN . Three‐dimensional printing in cardiovascular medicine: clinical applications and technological advancements. J Cardiovasc Transl Res. 2026;19(1):8:8. doi:10.1007/s12265-025-10733-9 41639493

[12] Wang DD , Qian Z , Vukicevic M , et al. 3D printing, computational modeling, and artificial intelligence for structural heart disease. JACC Cardiovasc Imaging. 2021;14(1):41‐60. doi:10.1016/j.jcmg.2019.12.022 32861647

[13] Xenofontos P , Zamani R , Akrami M . The application of 3D printing in preoperative planning for transcatheter aortic valve replacement: a systematic review. Biomed Eng Online. 2022;21(1):59. doi:10.1186/s12938-022-01029-z PMC9434927 36050722

[14] Hahn RT , Lindenfeld J , Lim SD , Mack MJ , Burkhoff D . Structural cardiac interventions in patients with heart failure: JACC scientific statement. J Am Coll Cardiol. 2024;84(9):832‐847. doi:10.1016/j.jacc.2024.05.061 39168570

[15] Thangaraj PM , Benson SH , Oikonomou EK , Asselbergs FW , Khera R . Cardiovascular care with digital twin technology in the era of generative artificial intelligence. Eur Heart J. 2024;45:4808‐4821. doi:10.1093/eurheartj/ehae619 PMC11638093 39322420

[16] Gu F , Meyer AJ , Ježek F , et al. Identification of digital twins to guide interpretable AI for diagnosis and prognosis in heart failure. NPJ Digit Med. 2025;8(1):110. doi:10.1038/s41746-025-01501-9 PMC11836287 39966509

[17] Sel K , Hawkins‐Daarud A , Chaudhuri A , et al. Survey and perspective on verification, validation, and uncertainty quantification of digital twins for precision medicine. NPJ Digit Med. 2025;8(1):40. doi:10.1038/s41746-025-01447-y PMC11742391 39825103

[18] Geddes JR , Jensen CW , Tanade C , et al. Digital twins for noninvasively measuring predictive markers of right heart failure. NPJ Digit Med. 2025;8(1):545. doi:10.1038/s41746-025-01920-8 PMC12379216 40855169

[19] Topol EJ . High‐performance medicine: the convergence of human and artificial intelligence. Nat Med. 2019;25(1):44‐56. doi:10.1038/s41591-018-0300-7 30617339

[20] Haddad S , Karzoun AA , Raffoul L , et al. Next‐gen heart solutions: 3D bioprinting and AI in valve replacement therapy. Ann 3D Print Med. 2025;20:100219. doi:10.1016/j.stlm.2025.100219

[21] Ma Y , Ding P , Li L , et al. Three‐dimensional printing for heart diseases: clinical application review. Bio‐Des Manuf. 2021;4(3):675‐687. doi:10.1007/s42242-021-00125-8 PMC8085656 33948306

[22] Bamford P , Abdelrahman A , Malkin CJ , Cunnington MS , Blackman DJ , Ali N . Artificial intelligence in heart valve disease: diagnosis, innovation and treatment. A State‐of‐the‐Art Review Br J Cardiol. 2024;31(3):31. doi:10.5837/bjc.2024.031 PMC11795922 39917554

[23] Park MH , Zhu Y , Imbrie‐Moore AM , et al. Heart valve biomechanics: the frontiers of modeling modalities and the expansive capabilities of ex vivo heart simulation. Front Cardiovasc Med. 2021;8:673689. doi:10.3389/fcvm.2021.673689 PMC8295480 34307492

[24] Agarwal A , Ranard L , Vahl T , Khalique O . Advanced computer simulation based on cardiac imaging in planning of structural heart disease interventions. J Clin Med. 2025;14(19):6885. doi:10.3390/jcm14196885 PMC12525038 41095965

[25] Fan Y , Yang Z , Li Q , et al. Heart failure: epidemiology, pathophysiology, and management. MedComm. 2026;7(3):e70625. doi:10.1002/mco2.70625 PMC13042786 41929360

[26] Zhang W , Motiwale S , Hsu MC , Sacks MS . Simulating the time evolving geometry, mechanical properties, and fibrous structure of bioprosthetic heart valve leaflets under cyclic loading. J Mech Behav Biomed Mater. 2021;123:104745. doi:10.1016/j.jmbbm.2021.104745 PMC8482999 34482092

[27] Whelan A , Williams E , Fitzpatrick E , et al. Collagen fibre‐mediated mechanical damage increases calcification of bovine pericardium for use in bioprosthetic heart valves. Acta Biomater. 2021;128:384‐392. doi:10.1016/j.actbio.2021.04.046 33945880

[28] Suri RM , Edris A , Hasan F . New ways of thinking about senescent bioprosthetic heart valve therapy. JACC Cardiovasc Interv. 2015;8(13):1745‐1747. doi:10.1016/j.jcin.2015.09.009 26585624

[29] Kostyunin AE , Glushkova TV , Lobov AA , et al. Proteolytic degradation is a major contributor to bioprosthetic heart valve failure. J Am Heart Assoc. 2023;12(1):e028215. doi:10.1161/JAHA.122.028215 PMC9973599 36565196

[30] Sacks MS . A review on the biomechanical effects of fatigue on the porcine bioprosthetic heart valve. J Long‐Term Eff Med Implants. 2017;27(2–4):181‐197. doi:10.1615/JLongTermEffMedImplants.v27.i2-4.60 29773039

[31] Martin C , Sun W . Comparison of transcatheter aortic valve and surgical bioprosthetic valve durability: a fatigue simulation study. J Biomech. 2015;48(12):3026‐3034. doi:10.1016/j.jbiomech.2015.07.031 PMC4592817 26294354

[32] Soares JS , Feaver KR , Zhang W , Kamensky D , Aggarwal A , Sacks MS . Biomechanical behavior of bioprosthetic heart valve heterograft tissues: characterization, simulation, and performance. Cardiovasc Eng Technol. 2016;7(4):309‐351. doi:10.1007/s13239-016-0276-8 PMC5537391 27507280

[33] Bagga S , Chhatriwalla AK . Updates on bioprosthetic valve fracture. Expert Rev Cardiovasc Ther. 2023;21(6):409‐422. doi:10.1080/14779072.2023.2217356 37222483

[34] Patel PM , Zapata D , Qu W , et al. Bioprosthetic pulmonary valve dysfunction in congenital heart disease. Ann Thorac Surg. 2023;115(3):641‐648. doi:10.1016/j.athoracsur.2021.12.025 35031286

[35] Lee JH , Rygg AD , Kolahdouz EM , et al. Fluid‐structure interaction models of bioprosthetic heart valve dynamics in an experimental pulse duplicator. Ann Biomed Eng. 2020;48(5):1475‐1490. doi:10.1007/s10439-020-02466-4 PMC7154025 32034607

[36] Koziarz A , Makhdoum A , Butany J , Ouzounian M , Chung J . Modes of bioprosthetic valve failure: a narrative review. Curr Opin Cardiol. 2020;35(2):123‐132. doi:10.1097/HCO.0000000000000711 31972604

[37] Zhang W , Sacks MS . Modeling the response of exogenously crosslinked tissue to cyclic loading: the effects of permanent set. J Mech Behav Biomed Mater. 2017;75:336‐350. doi:10.1016/j.jmbbm.2017.07.013 PMC5614899 28780254

[38] Whelan A , O'Brien G , Szagdaj A , O'Reilly D , Lally C . Investigation into early stage fatigue‐damage accumulation in glutaraldehyde‐fixed bovine pericardium using a novel equibiaxial bulge inflation system. J Mech Behav Biomed Mater. 2021;121:104588. doi:10.1016/j.jmbbm.2021.104588 34091153

[39] Tsolaki E , Corso P , Zboray R , et al. Multiscale multimodal characterization and simulation of structural alterations in failed bioprosthetic heart valves. Acta Biomater. 2023;169:138‐154. doi:10.1016/j.actbio.2023.07.044 37517619

[40] Amstrup Funder J , Christiansen R , Græsvig Sandahl O , Hjortdal V , Nygaard H , Michael Hasenkam J . Subcoronary stentless aortic valves are not superior to supra‐annular stented valves regarding turbulent stress. J Heart Valve Dis. 2015;24(6):722‐728. 27997778

[41] Nappi F , Mazzocchi L , Timofeva I , et al. A finite element analysis study from 3D CT to predict transcatheter heart valve thrombosis. Diagnostics (Basel). 2020;10(4):183. doi:10.3390/diagnostics10040183 PMC7235717 32225097

[42] Whiting R , Sander E , Conway C , Vaughan TJ . In silico modelling of aortic valve implants‐predicting in vitro performance using finite element analysis. J Med Eng Technol. 2022;46(3):220‐230. doi:10.1080/03091902.2022.2026506 35072595

[43] Anwar S , Singh GK , Varughese J , et al. 3D printing in complex congenital heart disease: across a spectrum of age, pathology, and imaging techniques. JACC Cardiovasc Imaging. 2017;10(8):953‐956. doi:10.1016/j.jcmg.2016.03.013 27450874

[44] Vukicevic M , Mosadegh B , Min JK , Little SH . Cardiac 3D printing and its future directions. JACC Cardiovasc Imaging. 2017;10(2):171‐184. doi:10.1016/j.jcmg.2016.12.001 PMC5664227 28183437

[45] Premyodhin N , Mandair D , Ferng AS , et al. 3D printed mitral valve models: affordable simulation for robotic mitral valve repair. Interact Cardiovasc Thorac Surg. 2018;26(1):71‐76. doi:10.1093/icvts/ivx243 PMC7868959 29049538

[46] Mahmood F , Owais K , Taylor C , et al. Three‐dimensional printing of mitral valve using echocardiographic data. JACC Cardiovasc Imaging. 2015;8(2):227‐229. doi:10.1016/j.jcmg.2014.06.020 25457770

[47] Didagelos M , Friderikos O , Ziakas A , et al. Mitral valve geometrical echocardiographic analysis and 3D computational modeling of a normal mitral valve. Futur Cardiol. 2023;19(9):453‐467. doi:10.2217/fca-2021-0157 37815033

[48] Olivieri LJ , Krieger A , Loke YH , Nath DS , Kim PC , Sable CA . Three‐dimensional printing of intracardiac defects from three‐dimensional echocardiographic images: feasibility and relative accuracy. J Am Soc Echocardiogr. 2015;28(4):392‐397. doi:10.1016/j.echo.2014.12.016 25660668

[49] Vukicevic M , Puperi DS , Jane Grande‐Allen K , Little SH . 3D printed modeling of the mitral valve for catheter‐based structural interventions. Ann Biomed Eng. 2017;45(2):508‐519. doi:10.1007/s10439-016-1676-5 27324801

[50] Fábián A , Lakatos BK , Tokodi M , et al. Geometrical remodeling of the mitral and tricuspid annuli in response to exercise training: a 3‐D echocardiographic study in elite athletes. Am J Physiol Heart Circ Physiol. 2021;320(5):H1774‐H1785. doi:10.1152/ajpheart.00877.2020 33666507

[51] Gosnell J , Pietila T , Samuel BP , Kurup HK , Haw MP , Vettukattil JJ . Integration of computed tomography and three‐dimensional echocardiography for hybrid three‐dimensional printing in congenital heart disease. J Digit Imaging. 2016;29(6):665‐669. doi:10.1007/s10278-016-9879-8 PMC5114226 27072399

[52] Rashmitha MKN , Kulkarni A , Kulkarni V . Segmentation and volumetric analysis of heart from cardiac CT images. Cardiovasc Eng Technol. 2024;15(4):383‐393. doi:10.1007/s13239-024-00715-4 38689094

[53] Angellotti D , Kampaktsis PΝ , Sticchi A , et al. CT assessment of right heart anatomy across tricuspid regurgitation severity grades: implications for transcatheter interventions. Int J Cardiovasc Imaging. 2025;41(10):2013‐2023. doi:10.1007/s10554-025-03505-8 PMC12491369 40900200

[54] Uccheddu F , Gallo M , Nocerino E , et al. Cardiovascular interventions planning through a three‐dimensional printing patient‐specific approach. J Cardiovasc Med (Hagerstown). 2019;20(9):584‐596. doi:10.2459/JCM.0000000000000832 31369473

[55] Hussein N , Honjo O , Barron DJ , Yoo SJ . Supravalvular aortic stenosis repair: surgical training of 2 repair techniques using 3D‐printed models. Interact Cardiovasc Thorac Surg. 2021;33(6):966‐968. doi:10.1093/icvts/ivab198 PMC8669556 34378022

[56] Maragiannis D , Jackson MS , Igo SR , et al. Replicating patient‐specific severe aortic valve stenosis with functional 3D modeling. Circ Cardiovasc Imaging. 2015;8(10):e003626. doi:10.1161/CIRCIMAGING.115.003626 26450122

[57] Giugno L , Faccini A , Carminati M . Percutaneous pulmonary valve implantation. Korean Circ J. 2020;50(4):302‐316. doi:10.4070/kcj.2019.0291 PMC7067602 32157831

[58] Anagnostopoulos S , Baltayiannis N , Koletsis NE , et al. 3D printing in medicine: bridging imaging, education, and practice. Arch Med Sci Atheroscler Dis. 2025;10:e172‐e188. doi:10.5114/amsad/209721 PMC12550675 41142700

[59] Marconi S , Lanzarone E , van Bogerijen GHW , et al. A compliant aortic model for in vitro simulations: design and manufacturing process. Med Eng Phys. 2018;59:21‐29. doi:10.1016/j.medengphy.2018.04.022 30077485

[60] Kurenov SN , Ionita C , Sammons D , Demmy TL . Three‐dimensional printing to facilitate anatomic study, device development, simulation, and planning in thoracic surgery. J Thorac Cardiovasc Surg. 2015;149(4):973‐979. doi:10.1016/j.jtcvs.2014.12.059 25659851

[61] Edelman RR , Serhal A , Pursnani A , Pang J , Koktzoglou I . Cardiovascular cine imaging and flow evaluation using fast interrupted steady‐state (FISS) magnetic resonance. J Cardiovasc Magn Reson. 2018;20(1):12. doi:10.1186/s12968-018-0433-3 PMC5819298 29458384

[62] Martínez Raya A , Aranda‐Ruiz J , Sal‐Anglada G , Jaureguizar SM , Braun M . Effect of printing orientation on the mechanical properties of low‐force stereolithography‐manufactured durable resin. Appl Sci. 2024;14(20):9529.

[63] Raya AM , Braun M , Carrasco‐Garrido C , González‐Albuixech VF . A frugal approach toward modeling of defects in metal 3D printing through statistical methods in finite element analysis. Computation. 2025;13(2):35.

[64] Illi J , Bernhard B , Nguyen C , et al. Translating imaging into 3D printed cardiovascular phantoms: a systematic review of applications, technologies, and validation. JACC Basic Transl Sci. 2022;7(10):1050‐1062. doi:10.1016/j.jacbts.2022.01.002 PMC9626905 36337920

[65] Hester RL , Pruett W , Clemmer J , Ruckdeschel A . Simulation of integrative physiology for medical education. Morphologie. 2019;103(343):187‐193. doi:10.1016/j.morpho.2019.09.004 PMC7219081 31563456

[66] Garekar S , Bharati A , Chokhandre M , et al. Clinical application and multidisciplinary assessment of three dimensional printing in double outlet right ventricle with remote ventricular septal defect. World J Pediatr Congenit Heart Surg. 2016;7(3):344‐350. doi:10.1177/2150135116645604 27142402

[67] Kiraly L , Tofeig M , Jha NK , Talo H . Three‐dimensional printed prototypes refine the anatomy of post‐modified Norwood‐1 complex aortic arch obstruction and allow presurgical simulation of the repair. Interact Cardiovasc Thorac Surg. 2016;22(2):238‐240. doi:10.1093/icvts/ivv320 26590304

[68] Hopfner C , Jakob A , Tengler A , et al. Design and 3D printing of variant pediatric heart models for training based on a single patient scan. 3D Print Med. 2021;7(1):25. doi:10.1186/s41205-021-00116-6 PMC8406574 34463879

[69] Farooqi KM , Cooper C . 3D printing and heart failure: the present and the future. JACC Heart Fail. 2019;7(2):132‐142. doi:10.1016/j.jchf.2018.09.011 30553901

[70] Aranda‐Michel E , Waldman LK , Trumble DR . Left ventricular simulation of cardiac compression: hemodynamics and regional mechanics. PLoS One. 2019;14(10):e0224475. doi:10.1371/journal.pone.0224475 PMC6822737 31671155

[71] Aroney N , Putrino A , Scalia G , Walters D . 3D printed cardiac fistula: guiding percutaneous structural intervention. Catheter Cardiovasc Interv. 2018;92(7):E478‐E480. doi:10.1002/ccd.27786 30260091

[72] Qian Z , Wang K , Liu S , et al. Quantitative prediction of paravalvular leak in Transcatheter aortic valve replacement based on tissue‐mimicking 3D printing. JACC Cardiovasc Imaging. 2017;10(7):719‐731. doi:10.1016/j.jcmg.2017.04.005 28683947

[73] Farooqi KM , Saeed O , Zaidi A , et al. 3D printing to guide ventricular assist device placement in adults with congenital heart disease and heart failure. JACC. Heart Failure. 2016;4(4):301‐311. doi:10.1016/j.jchf.2016.01.012 27033018

[74] Javan R , Herrin D , Tangestanipoor A . Understanding spatially complex segmental and branch anatomy using 3D printing: liver, lung, prostate, coronary arteries, and circle of Willis. Acad Radiol. 2016;23(9):1183‐1189. doi:10.1016/j.acra.2016.04.010 27283072

[75] Russ M , O'Hara R , Setlur Nagesh SV , et al. Treatment planning for image‐guided neuro‐vascular interventions using patient‐specific 3D printed phantoms. Proc SPIE Int Soc Opt Eng. 2015;9417:941726. doi:10.1117/12.2081997 PMC4712717 26778878

[76] Canning C , Guo J , Narang A , Thomas JD , Ahmad FS . The emerging role of artificial intelligence in Valvular heart disease. Heart Fail Clin. 2023;19(3):391‐405. doi:10.1016/j.hfc.2023.03.001 PMC11267973 37230652

[77] Safdar S , Zafar S , Zafar N , Khan NF . Machine learning based decision support systems (DSS) for heart disease diagnosis: a review. Artif Intell Rev. 2018;50:597‐623. doi:10.1007/s10462-017-9552-8

[78] Zhou Z , Xie K , Huang Y , et al. Automatic diagnosis of left valvular heart disease based on artificial intelligence stethoscope. JACC Adv. 2025;4(11 Pt 2):101993. doi:10.1016/j.jacadv.2025.101993 PMC12717554 40838911

[79] Thalappillil R , Datta P , Datta S , et al. Artificial intelligence for the measurement of the aortic valve annulus. J Cardiothorac Vasc Anesth. 2020;34(1):65‐71. doi:10.1053/j.jvca.2019.06.017 31351874

[80] Toggweiler S , Wyler von Ballmoos MC , Moccetti F , et al. A fully automated artificial intelligence‐driven software for planning of transcatheter aortic valve replacement. Cardiovasc Revasc Med. 2024;65:25‐31. doi:10.1016/j.carrev.2024.03.008 38467531

[81] Hernandez‐Suarez DF , Kim Y , Villablanca P , et al. Machine learning prediction models for in‐hospital mortality after transcatheter aortic valve replacement. JACC Cardiovasc Interv. 2019;12(14):1328‐1338. doi:10.1016/j.jcin.2019.06.013 PMC6650265 31320027

[82] Daniel PO , Gada H , Sotelo MR , et al. Enhanced detection of heart valve disease using integrated artificial intelligence at scale. Ann Thorac Surg. 2022;113(5):1499‐1504. doi:10.1016/j.athoracsur.2021.04.106 34139187

[83] Misumi Y , Miyagawa S , Yoshioka D , et al. Prediction of aortic valve regurgitation after continuous‐flow left ventricular assist device implantation using artificial intelligence trained on acoustic spectra. J Artif Organs. 2021;24(2):164‐172. doi:10.1007/s10047-020-01243-3 PMC8154812 33537860

[84] Voigt JU , Cvijic M . 2‐and 3‐dimensional myocardial strain in cardiac health and disease. JACC Cardiovasc Imaging. 2019;12(9):1849‐1863. doi:10.1016/j.jcmg.2019.01.044 31488253

[85] Hoang P , Guccione J . Finite element modeling in left ventricular cardiac biomechanics: from computational tool to clinical practice. Bioengineering (Basel). 2025;12(9):913. doi:10.3390/bioengineering12090913 PMC12467504 41007158

[86] Zhang Y , Wang VY , Morgan AE , et al. A novel MRI‐based finite element modeling method for calculating myocardial ischemia effect in patients with functional mitral regurgitation. Front Physiol. 2020;11:158. doi:10.3389/fphys.2020.00158 PMC7082816 32231584

[87] Sohn S , Momtahan N , Stevens LM , et al. Modulating hydrogel stiffness through light‐based 3D printing to mimic cardiac fibrosis and cardiomyocyte dysfunction using hiPSC‐derived cells. J Biomed Mater Res A. 2025;113(10):e37997. doi:10.1002/jbm.a.37997 41055580

[88] Watanabe T , Hatayama N , Guo M , Yuhara S , Shinoka T . Bridging the gap: advances and challenges in heart regeneration from in vitro to in vivo applications. Bioengineering (Basel). 2024;11(10):954. doi:10.3390/bioengineering11100954 PMC11505552 39451329

[89] Garcia‐Canadilla P , Sanchez‐Martinez S , Martí‐Castellote PM , et al. Machine‐learning‐based exploration to identify remodeling patterns associated with death or heart‐transplant in pediatric‐dilated cardiomyopathy. J Heart Lung Transplant. 2022;41(4):516‐526. doi:10.1016/j.healun.2021.11.020 35063339

[90] Lang FM , Lee BC , Lotan D , Sabuncu MR , Topkara VK . Role of artificial intelligence and machine learning to create predictors, enhance molecular understanding, and implement purposeful programs for myocardial recovery. Methodist Debakey Cardiovasc J. 2024;20(4):76‐87. doi:10.14797/mdcvj.1392 PMC11342843 39184156

[91] Mele M , Imbrici P , Mele A , et al. Short‐term anti‐remodeling effects of gliflozins in diabetic patients with heart failure and reduced ejection fraction: an explainable artificial intelligence approach. Front Pharmacol. 2023;14:1175606. doi:10.3389/fphar.2023.1175606 PMC10289166 37361206

[92] Wu D , Ono R , Wang S , Kobayashi Y , Sughimoto K , Liu H . Pulse wave signal‐driven machine learning for identifying left ventricular enlargement in heart failure patients. Biomed Eng Online. 2024;23(1):60. doi:10.1186/s12938-024-01257-5 PMC11193305 38909231

[93] Eggertsen TG , Travers JG , Hardy EJ , Wolf MJ , McKinsey TA , Saucerman JJ . Logic‐based machine learning predicts how escitalopram attenuates cardiomyocyte hypertrophy. Proc Natl Acad Sci USA. 2025;122(10):e2420499122. doi:10.1073/pnas.2420499122 PMC11912418 40035765

[94] Sapna F , Raveena F , Chandio M , et al. Advancements in heart failure management: a comprehensive narrative review of emerging therapies. Cureus. 2023;15(10):e46486. doi:10.7759/cureus.46486 PMC10623504 37927716

[95] Babaei H , Mendiola EA , Neelakantan S , et al. A machine learning model to estimate myocardial stiffness from EDPVR. Sci Rep. 2022;12(1):5433. doi:10.1038/s41598-022-09128-6 PMC8971532 35361836

[96] Barrado‐Ballestero S , Yttergren ST , Hahn M , et al. Deep learning‐enhanced 3D imaging unveils semaglutide impact on cardiac fibrosis. Br J Pharmacol. 2026;183(5):1030‐1047. doi:10.1111/bph.70217 41121520

[97] Ward M , Yeganegi A , Baicu CF , et al. Ensemble machine learning model identifies patients with HFpEF from matrix‐related plasma biomarkers. Am J Physiol Heart Circ Physiol. 2022;322(5):H798‐H805. doi:10.1152/ajpheart.00497.2021 PMC8993521 35275763

[98] Doehner W , Boriani G , Potpara T , et al. Atrial fibrillation burden in clinical practice, research, and technology development: a clinical consensus statement of the European Society of Cardiology Council on stroke and the European heart rhythm association. Europace. 2025;27(3):euaf019. doi:10.1093/europace/euaf019 PMC11901050 40073206

[99] Koopsen T , Gerrits W , van Osta N , et al. Virtual pacing of a patient's digital twin to predict left ventricular reverse remodelling after cardiac resynchronization therapy. Europace. 2024;26(1):euae009. doi:10.1093/europace/euae009 PMC10825733 38288616

[100] Sankarankutty AC , Greiner J , Bragard J , et al. Etiology‐specific remodeling in ventricular tissue of heart failure patients and its implications for computational modeling of electrical conduction. Front Physiol. 2021;12:730933. doi:10.3389/fphys.2021.730933 PMC8523803 34675817

[101] Lechuga CG , Kachabi A , Colebank MJ , Korcarz CE , Raza F , Chesler NC . Patient‐specific lumped‐parameter model for quantifying vessel‐specific remodeling and predicting right ventricular function in pulmonary hypertension. Compr Physiol. 2026;16(1):e70102. doi:10.1002/cph4.70102 PMC12835583 41588726

[102] Hamid N , Jorde UP , Reisman M , et al. Transcatheter left ventricular restoration in patients with heart failure. J Card Fail. 2023;29(7):1046‐1055. doi:10.1016/j.cardfail.2023.03.003 36958391

[103] Bath AS , Raja J , Maturana MA , Khouzam RN . Complex interventions in the failing heart. Curr Opin Organ Transplant. 2021;26(3):267‐272. doi:10.1097/MOT.0000000000000877 33938462

[104] Mody R , Nee Sheth AB , Dash D , et al. Device therapies for heart failure with reduced ejection fraction: a new era. Front Cardiovasc Med. 2024;11:1388232. doi:10.3389/fcvm.2024.1388232 PMC11527719 39494238

[105] Pancaldi E , Benenati S , Emdin M , et al. Dispositivi transcatetere per l'insufficienza cardiaca cronica: stato dell'arte [Transcatheter devices for chronic heart failure: state of the art]. G Ital Cardiol (Rome). 2022;23(12 Suppl 2):5S‐18S. doi:10.1714/3949.39287 36636870

